# Immunotherapy resistance in non-small cell lung cancer: from mechanisms to therapeutic opportunities

**DOI:** 10.1186/s13046-025-03519-z

**Published:** 2025-08-23

**Authors:** Huiyu Wang, Xiaomin Niu, Zhenning Jin, Shaoxing Zhang, Rong Fan, Hua Xiao, Shen S. Hu

**Affiliations:** 1 Zhejiang Key Laboratory of Multiomics and Molecular Enzymology, Yangtze Delta Region Institute of Tsinghua University, Zhejiang, 314006 China; 2 Department of Biotechnology and Biomedicine, Yangtze Delta Region Institute of Tsinghua University, Zhejiang, 314006 China; 3 Department of Shanghai Lung Cancer Center, Shanghai Chest Hospital, Shanghai Jiao Tong University School of Medicine, Shanghai, 200030 China; 4 Shanghai Key Laboratory of Thoracic Tumor Biotherapy, Shanghai, 200030 China; 5 State Key Laboratory of Microbial Metabolism, Joint International Research Laboratory of Metabolic & Developmental Sciences, School of Life Sciences and Biotechnology, Shanghai Jiao Tong University, Shanghai, 200240 China

**Keywords:** Lung cancer, Immunotherapy, ICIs, Immune resistance, Combination therapy

## Abstract

This review provides a comprehensive synthesis of current knowledge on immunotherapy resistance in non-small cell lung cancer (NSCLC), a disease that accounts for approximately 85% of all lung cancer cases and remains the leading cause of cancer-related death worldwide. Although immune checkpoint inhibitors (ICIs) have significantly improved survival for a subset of patients with advanced NSCLC, over 70% of cases ultimately exhibit primary or acquired resistance, underscoring the urgent need to understand the underlying mechanisms. The review categorizes resistance into tumor-intrinsic and tumor-extrinsic processes and provides an in-depth mechanistic analysis of how factors such as tumor antigen loss, impaired antigen presentation, cGAS-STING pathway dysregulation, metabolic reprogramming in tumor microenvironment (TME), immune cell exhaustion, and microbiomes collectively contribute to immune escape. In parallel, the influence of the lung and gut microbiome on shaping immunotherapy responses is discussed, with emphasis on microbial dysbiosis, immunosuppressive metabolite production, and TME remodeling. Therapeutic strategies to overcome resistance are also discussed, including combination approaches involving chemotherapy, radiotherapy, and antiangiogenic agents, as well as epigenetic modulators (HDAC and BET inhibitors). Moreover, the review explores bispecific antibodies, antibody-drug conjugates, and small-molecule agents that enhance T cell function or disrupt immunosuppressive signaling networks. By integrating insights from preclinical models and clinical trials, the review underscores the necessity of biomarker-guided patient stratification, combination immunotherapy approaches, and interventions that restore tumor immunogenicity. It concludes that a multipronged therapeutic strategy, one that addresses both immune evasion and TME-induced suppression, holds the greatest promise for improving response durability and advancing personalized immunotherapy for NSCLC.

## Background

**Fig. 1:**
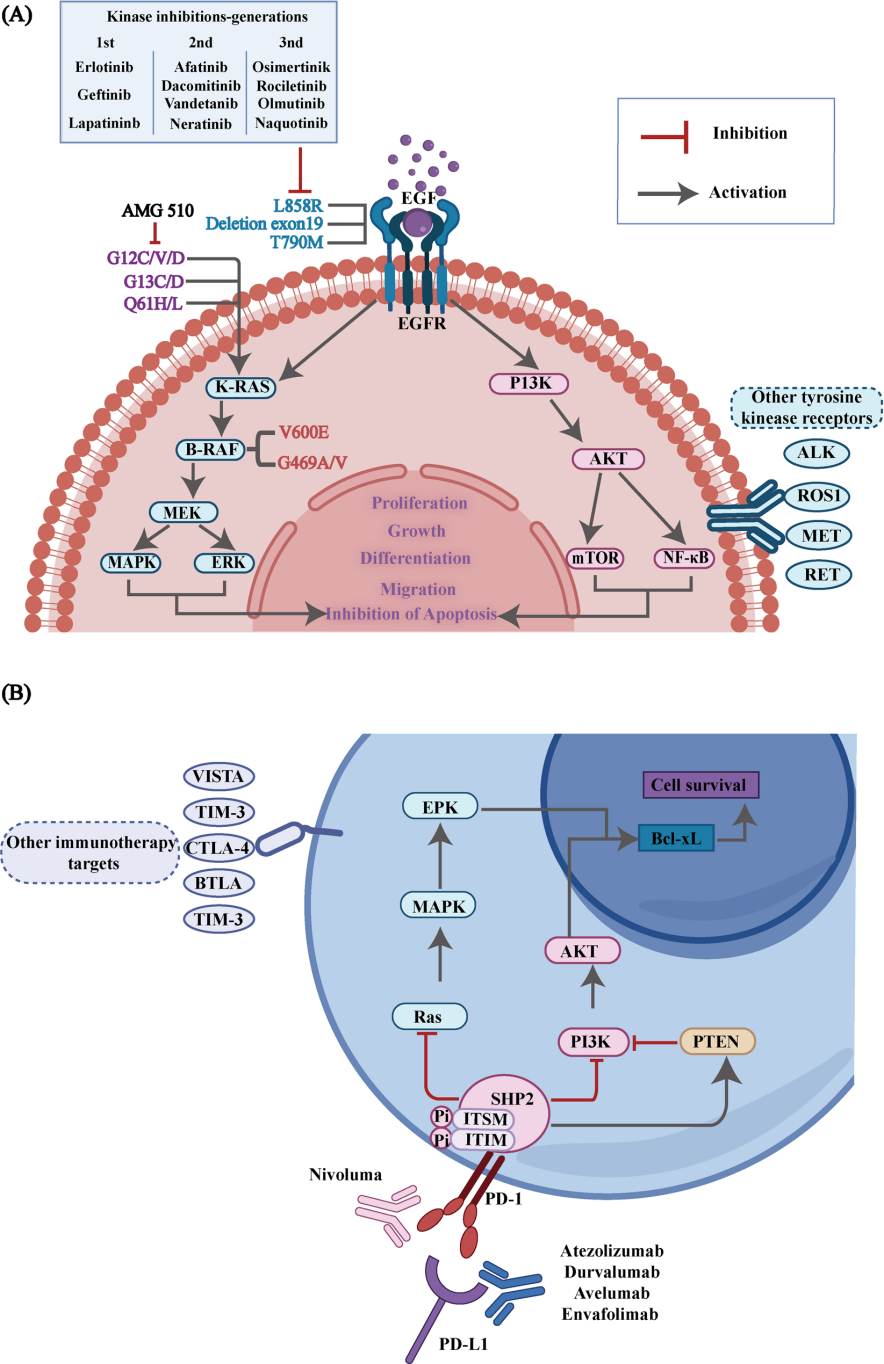
Pathogenic Cell Receptors and Relevant Signaling Pathways Involved in Targeted Therapies and Immunotherapies for NSCLC. (**A**) EGFR belongs to the ErbB family of receptor tyrosine kinases (RTKs). Ligand binding to the extracellular domain induces conformational change, triggering tyrosine phosphorylation, receptor dimerization, and downstream signaling activation, ultimately driving cellular growth and survival. (**B**) PD-1/PD-L1 binding induces phosphorylation of immunoreceptor tyrosine-based switch motifs (ITSMs) and immunoreceptor tyrosine-based inhibitory motifs (ITIMs) within the intracellular domain of PD-1. This recruits Src homology 2 (SH2) domain-containing tyrosine phosphatase (SHP-2), which suppresses PI3K/AKT and Ras/MAPK/ERK signaling pathways, ultimately driving T-cell dysfunction and apoptosis

As the most common and lethal malignancies around the world, lung cancer poses a significant challenge to the health of the public according to worldwide cancer statistics [1, 2]. Non-small cell lung cancer (NSCLC) accounts for approximately 85% of all lung cancer cases and is the most dominant histological type of lung cancer [3, 4]. Treatment approaches for NSCLC have evolved from traditional surgery, radiotherapy (RT) and chemotherapy to more precise approaches like immunotherapy and targeted therapy [5–7]. For targeted therapy, the holistic molecular profiling of NSCLC has identified 10 genes that are most frequently mutated in lung cancer. These genes are tumor protein 53 (*TP53*), kirsten rat sarcoma viral oncogene homolog (*KRAS*), serine/threonine kinase 11 (*STK11*), epidermal growth factor receptor (*EGFR*), B-Raf proto-oncogene, serine/threonine kinase (*BRAF*), RNA binding motif protein 10 (*RBM10*), anaplastic lymphoma kinase *(ALK*), reactive oxygen species (*ROS*) proto-oncogene 1 (*ROS1*), mesenchymal to epithelial transition factor (*MET*) and RET proto-oncogene (*RET*). Currently, *EGFR* and *KRAS* are the most established targets with approved therapies in clinical practice (Fig. 1A) [8]. In parallel, immunotherapy has emerged as an essential treatment modality, with a focus on modulating immune checkpoint (ICP) molecules [9–11]. These molecules play a critical role in regulating the immune system of the body and prevent excessive immune activation and potential autoimmune reactions [12, 13]. They include galectin-9 (Gal-9), programmed death-ligand 1 (PD-L1), programmed death-1 (PD-1), cytotoxic T-lymphocyte-associated protein 4 (CTLA-4), B and T lymphocyte attenuator (BTLA), T-cell immunoglobulin and mucin-domain containing-3 (TIM-3), lymphocyte activation gene-3 (LAG-3), T-cell immunoreceptor with Ig and ITIM domains (TIGIT) and V-type immunoglobulin domain-containing suppressor of T-cell activation (VISTA). However, these immune checkpoint pathways can be exploited by tumors to evade immune surveillance. In the presence of overexpressed or hyperactivated immune checkpoint molecules, the immune system becomes suppressed, which enables cancer cells to avoid detection and clearance and therefore accelerates tumor progression [14]. The PD-1/PD-L1 signaling pathway, one of the earliest immune checkpoints identified, is quite important in immune evasion. Protein-protein interactions realize the binding of PD-L1 to PD-1 receptors on T cells, which reduces immune responses against nearby normal tissues. This mechanism is conducive to preventing autoimmune damage under normal physiological conditions. In tumors, however, aberrant overexpression of PD-L1 suppresses T cell activity and weakens antitumor immune responses, thereby allowing cancer cells to evade immune recognition and elimination. (Fig. 1B) [15].

This immune escape mechanism has prompted the development of multiple immune checkpoint inhibitors (ICIs) targeting the PD-1/PD-L1 signaling axis, many of which have been approved for clinical use (Table 1). In 2015, the food and drug administration (FDA) approved Nivolumab for the treatment of metastatic NSCLC in patients who had progressed following platinum-based chemotherapy, particularly those with squamous histology [16, 17]. Pembrolizumab was subsequently approved in the same year for the treatment of advanced NSCLC patients with PD-L1 expression and was later extended to first-line therapy in patients with high PD-L1 expression [18, 19]. The phase III KEYNOTE-024 trial demonstrated the superiority of pembrolizumab over platinum-based chemotherapy as a first-line monotherapy in NSCLC, showing a median overall survival (OS) of 30.0 vs. 14.2 months, and a 5-year survival rate of 31.9% vs. 16.3% [19]. In 2016, Atezolizumab was approved for the treatment of metastatic NSCLC in patients who had progressed after platinum-based chemotherapy. This indication was further expanded in 2020 to include first-line monotherapy for metastatic NSCLC patients with high PD-L1 expression (TC ≥ 50% or IC ≥ 10%) and without *EGFR* or *ALK* alterations [20]. In 2017, Durvalumab became the first ICI approved for unresectable stage III NSCLC, based on its demonstrated efficacy as consolidation therapy after concurrent chemoradiotherapy [21]. Although Avelumab has been primarily approved for Merkel cell carcinoma and other malignancies, clinical trials have indicated its potential efficacy in NSCLC. However, it has not yet received formal approval for this indication [22]. In 2022, Cemiplimab was approved in combination with platinum-based chemotherapy for the treatment of advanced NSCLC without diver gene mutations [23]. In China, Sintilimab was approved in 2021 by the National Medical Products Administration (NMPA) as a first-line treatment for advanced squamous NSCLC in combination with chemotherapy [24]. In 2024, Toripalimab was approved as part of a perioperative immuno-chemotherapy regimen for resectable stage IIIA-IIIB NSCLC [25]. Envafolimab, the first subcutaneously injectable PD-L1 antibody, has shown efficacy in advanced NSCLC when combined with chemotherapy or Endostar [26].

**Table 1:** Inhibitors of the PD-1/PD-L1 signaling pathway

Name	Target	Research stage	Indications
Nivolumab [16]	PD-1	On the market	NSCLC, melanoma, renal cell carcinoma, Hodgkin lymphoma, etc.
Pembrolizumab [18]	PD-1	On the market	NSCLC, melanoma, renal cell carcinoma, Hodgkin lymphoma, etc.
Cemiplimab [23]	PD-1	On the market	Cutaneous squamous cell carcinoma, NSCLC, basal cell carcinoma
Atezolizumab [20]	PD-L1	On the market	NSCLC, bladder cancer, triple-negative breast cancer, etc.
Avelumab [22]	PD-L1	On the market	Merkel cell carcinoma, urothelial carcinoma
Durvalumab [21]	PD-L1	On the market	NSCLC, bladder cancer, small cell lung cancer
Envafolimab [218]	PD-L1	In clinical trials	Soft tissue sarcoma, biliary tract cancer
Sintilimab [24]	PD-1	On the market	NSCLC, liver cancer, gastric cancer
Toripalimab [25]	PD-1	On the market	Nasopharyngeal carcinoma, melanoma, lung cancer

Notably, significant heterogeneity exists in the expression of PD-L1 across different subtypes of NSCLC, especially between lung squamous cell carcinoma (LUSC) and lung adenocarcinoma (LUAD), which has important implications for molecular characterization and immunotherapy strategies [27, 28]. In a large cohort of 3,185 Chinese NSCLC patients, high PD-L1 expression had a significantly greater proportion in LUSC (20.84%) than in LUAD (15.98%) [29]. This disparity may be partially ascribed to distinct underlying genetic landscapes. LUSC typically harbors a higher prevalence of smoking-related mutations like TP53 [30]. The IMpower131 trial demonstrated that atezolizumab combined with carboplatin and nab-paclitaxel provides both progression free survival (PFS) and OS benefits for patients with metastatic LUSC whose tumors exhibit high PD-L1 expression [31]. In contrast, LUAD more frequently carries oncogenic driver mutations in *EGFR*, *KRAS*, *ALK* or *ROS1*. LUAD with *STK11/LKB1* deletion demonstrates low PD-L1 expression, reduced T-cell infiltration, and neutrophil enrichment, leading to primary resistance to immunotherapy [32]. *EGFR* wild-type tumors are more likely to be PD-L1 positive compared to *EGFR* mutant tumors. *EGFR* mutations exhibit deficient T-cell infiltration and display an immunotolerant phenotype. This may partially explain why some *EGFR* mutant patients with PD-L1 positivity fail to respond to anti-PD-1/L1 therapy [33]. In the KEYNOTE-789 study, addition of pembrolizumab to chemotherapy in patients with TKI-resistant, *EGFR* mutant, metastatic nonsquamous NSCLC did not significantly prolong PFS or OS versus placebo plus chemotherapy [34]. *KRAS* mutations in LUAD, particularly *KRAS*-G12C, are closely associated with elevated PD-L1 expression and an immunosuppressive tumor microenvironment [35]. Mechanistically, *KRAS* activation drives PD-L1 transcription via the ERK/MAPK pathway, leading to T cell exhaustion and immune escape. In parallel, *KRAS*-driven tumors also exhibit increased secretion of immunosuppressive cytokines such as IL-10 and TGF-β, which facilitate the recruitment and activation of myeloid-derived suppressor cells (MDSCs) and regulatory T cells (Tregs). Additionally, in *KRAS* mutant LUAD, the tumor suppressors *LKB1/STK11* and *KEAP1* are frequently mutated, both of which have been shown to drive immune evasion. Several clinical studies have shown that NSCLC patients with *KRAS* mutations exhibit a more favorable response to ICB compared to patients with wild-type tumors [36]. Ongoing clinical trials, including NCT03600883, NCT04185883, NCT03785249, and NCT04699188, are actively investigating combination strategies involving *KRAS*-G12C inhibitors and anti-PD-1 therapies. In summary, the expression of PD-L1 and its regulatory mechanisms differ fundamentally between NSCLC subtypes owing to distinct genetic and immunologic contexts. A comprehensive evaluation that integrates PD-L1 levels and driver gene alterations may provide a more precise framework for stratifying patients and guiding personalized immunotherapy.

However, immunotherapy remains ineffective in a subset of patients. Data indicates that only 27–46% of patients respond to initial therapy with ICIs, and of those, up to 65% develop resistance within four years [37], which represents a key barrier to further improving outcomes in advanced lung cancer patients [38]. Recently, the Society for Immunotherapy of Cancer (SITC) defined distinct resistance patterns to anti-PD-1 and anti-PD-L1 therapies, like primary and acquired resistance [14]. Primary resistance refers to resistance occurring in cancer patients who do not profit from long-term exposure to ICIs. Acquired resistance, on the other hand, features preliminary benefit from immunotherapy and then the development of progressive disease [39].

The mechanisms underlying resistance to ICIs are multifactorial and involve intrinsic and extrinsic factors [40]. Tumor-intrinsic mechanisms include a low burden or poor quality of neoantigens, defects in antigen presentation, and aberrant regulation of immune-evasive signaling pathways such as cyclic guanylate (GMP)-adenosine monophosphate (AMP) synthase-stimulator of interferon genes (cGAS-STING) pathway. Tumor-extrinsic factors, such as the presence of immunosuppressive tumor microenvironment TME, the exhaustion of CD8⁺ T cells, and the influence of gut and lung microbiomes, also contribute to immune evasion. A deeper understanding of these mechanisms lays the foundation for developing personalized treatment strategies that target specific resistance pathways. To address these challenges, we further summarize emerging combination strategies designed to overcome immunotherapy resistance. These approaches offer promising avenues to re-sensitize tumors to ICIs and improve therapeutic outcomes in NSCLC.

## Mechanisms of resistance to immunotherapy

### Tumor-Intrinsic mechanisms

#### Quantity and quality of tumor antigens

As abnormal peptide fragments generated by somatic mutations, tumor neoantigens are specifically expressed on malignant cell surfaces, where they can be perceived as “non-self” by the immune system, which thereby triggers T cell-mediated immune attacks [41, 42]. Both the quantity and quality of neoantigens as critical targets for ICI therapy jointly determine the sensitivity of tumors to immunotherapy [43].

**Fig. 2:**
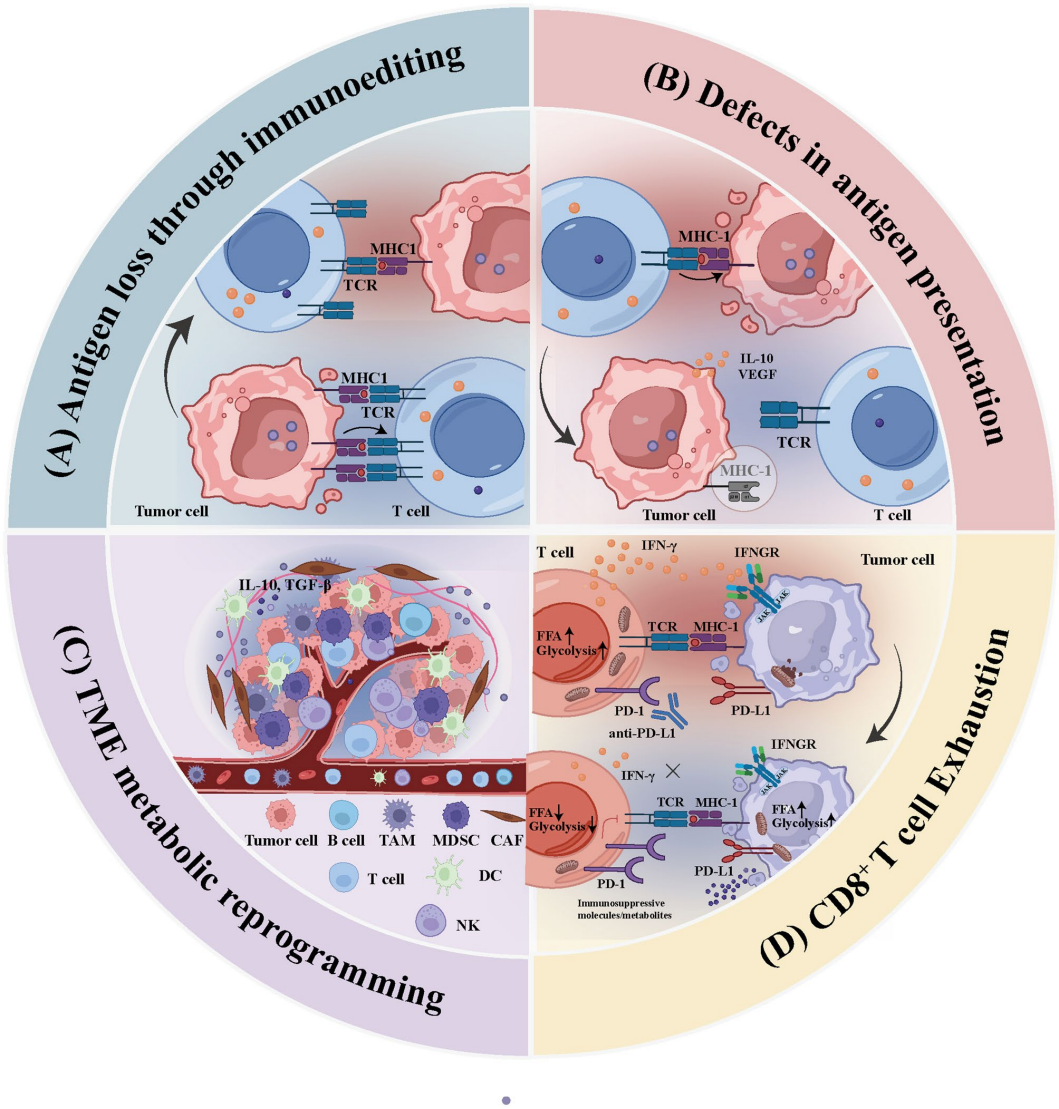
Schematic Diagram of the Mechanisms Underlying Resistance to Immunotherapy. (**A**) The immune insufficiency of tumor antigens leads to resistance to immunotherapy. (**B**) During the process of antigen presentation, multiple protein mutations make it impossible to process and present specific antigen proteins, which thus prevents the activation of effector T cells and leads to resistance to ICI therapy. (**C**) The exhaustion of T cells leads to decreased cytokine (e.g., IFN-γ) secretion and increased inhibitory receptor (e.g., PD-1 and TIM-3) expression, which reduces the efficacy of ICI therapy. (**D**) Resistance to immunotherapy caused by suppression within the TME

Clinical and mechanistic studies have shown that the loss of neoantigens is a central mechanism that underlies resistance to anti-PD-1/PD-L1 therapy. Tumor cells can selectively lose highly immunogenic neoantigens through a variety of mechanisms such as transcriptional repression, epigenetic silencing, copy number loss and post-translational modifications, which thereby evade immune recognition [44, 45]. Known as immunoediting, this process reflects the immune-driven de-immunization of tumors under selective immune pressure (Fig. 2A). In particular, if subclones within a tumor carry specific neoantigens readily subjected to recognition and elimination by CD8⁺ T cells, these subpopulations will be progressively eliminated. This leads to the reduced clonal expression of neoantigens, increased heterogeneity and ultimately the weakened long-term immune control of the tumor [46]. Even if certain mutations generate potential neoantigens, their functional expression may be silenced if encoding genes are repressed by epigenetic mechanisms like DNA methylation and histone modifications (like trimethylation of lysine 27 on histone H3 (H3K27me3)). This renders them undetectable to the immune system. Luksza et al. proposed a neoantigen fitness model that quantitatively ranks neoantigens. They demonstrated that tumors enriched with high-quality, clonally expressed neoantigens responded to checkpoint blockade immunotherapy more favorably. Conversely, immune evasion may arise through immunoediting and the loss of highly immunogenic neoantigens, which contributes to acquired resistance [43]. To sum up, the selective loss of neoantigens, increased expression heterogeneity, and reduced neoantigen quality collectively weaken the ability of the immune system to sustain tumor control and constitute the core mechanisms of immune resistance.

#### Defects in the antigen presentation process

**Fig. 3:**
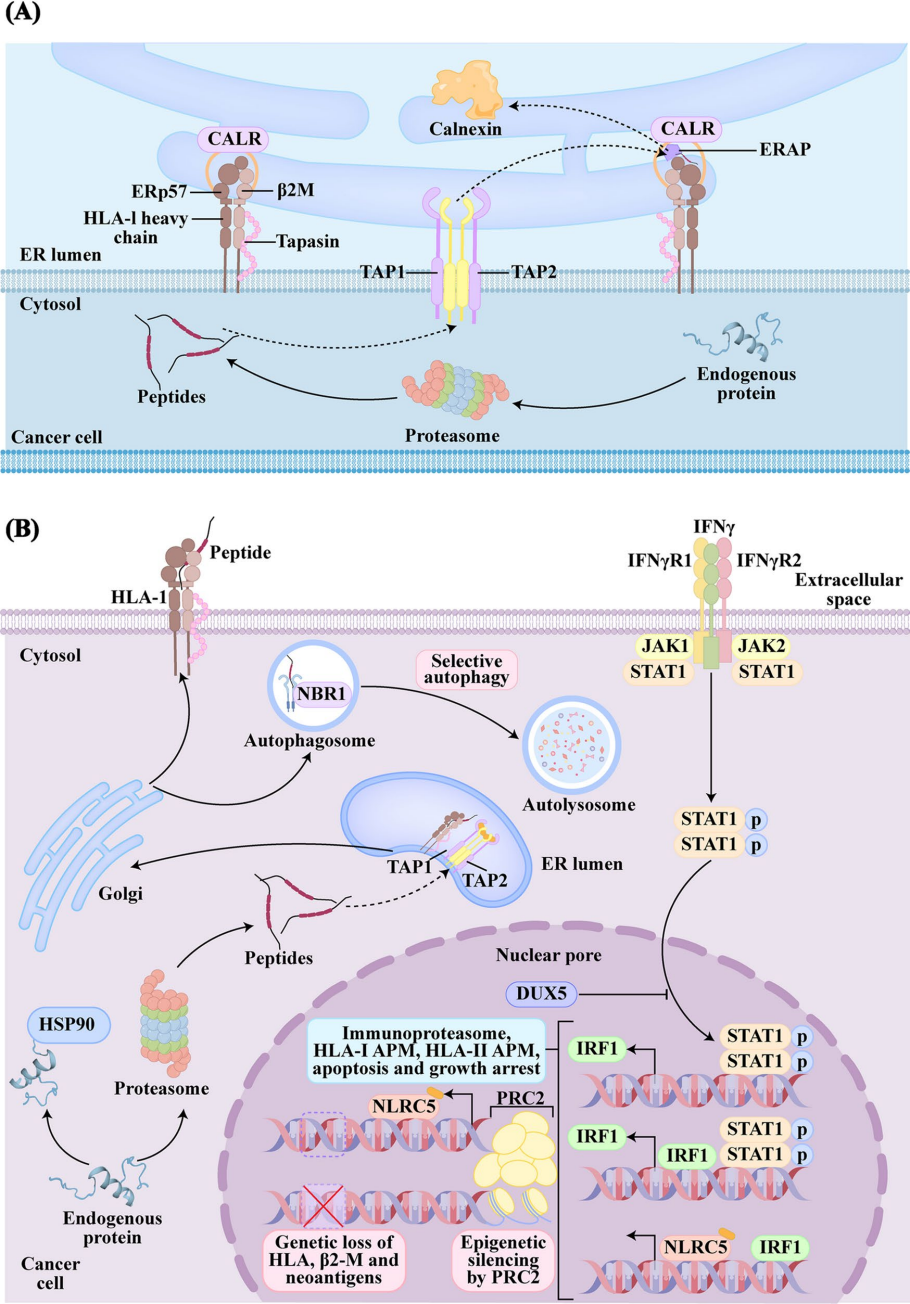
Antigen Presentation Pathway and Its Dysregulation in Tumors. (**A**) Antigen processing and presentation machinery. (**B**) Under inflammatory conditions, IFN-γ signaling induces immunoproteasome subunit expression and upregulates APM components, enhancing antigen presentation and promoting tumor cell apoptosis. IFN-γ signaling up-regulates APM components and immunoproteasome subunits, which enhances antigen presentation and induces tumor cell apoptosis. Tumor cells nevertheless circumvent immune surveillance through multiple resistance mechanisms

In tumor immunotherapy, defects or disruptions in the process of antigen presentation can prevent the immune system from effectively recognizing tumor cells, which triggers immune resistance [47]. The major histocompatibility complex class I (MHC-I)-mediated antigen presentation pathway plays a central role in this process. Normally, endogenous proteins are subjected to processing by the proteasome into peptides. The peptides are then translocated through the transporters associated with antigen processing (TAP) into the endoplasmic reticulum (ER) where they may get further trimmed by ER aminopeptidase (ERAP) either as free peptides or after loading onto MHC-I. Loading onto MHC-I is a multistep process facilitated by the peptide loading complex (PLC), which consists of the TAP and ER chaperones. First, calnexin promotes the initial folding and the assembly of MHC-I heavy chain, then the MHC-I heavy chain assembles with β2-m in the absence of a peptide. This empty complex is highly unstable for most MHC-I alleles, and it is stabilized by association with the core PLC consisting of the chaperone tapasin in complex with ERp57 and TAP, via calreticulin (CALR). A single TAP heterodimer associates with two empty MHC-I complexes. Tapasin then proofreads peptides for stable binding in the groove formed by the MHC-I heavy chain α_1_ and α_2_ domains. Binding of high-affinity peptide induces the dissociation of MHC-I complex from the PLC and subsequent trafficking to the cell surface (Fig. 3A).

However, tumors secrete IL-10, vascular endothelial growth factor (VEGF) and other inhibitory factors to promote the abnormal differentiation of myeloid cells, which leads to a reduction in antigen presenting cells (APCs)-related precursor dendritic cells (DCs) (Fig. 2B) [48]. Peripheral immature DCs lacking or expressing low levels of costimulatory molecules like CD80 and CD86 may not effectively activate naive T cells. Instead of inducing a functional effector T cell response, antigen presentation by these DCs when adequate co-stimulation is absent can lead to the anergy or tolerance of T cells, which promotes a regulatory or unresponsive phenotype [49]. Furthermore, tumors are capable of evading immune surveillance by interfering with antigen presentation machinery (APM) through multiple layers of regulatory mechanisms, which ultimately causes resistance against ICIs [50, 51]. The core mechanisms underlying such interference can be categorized as follows:

*Reduction of MHC-I Expression by Genetic Alterations. MHC-A/B/C* genes are located on chromosome 6. The loss of one allele can reduce MHC-I expression, while losing both alleles may result in complete absence [52]. β2-microglobul*in (β2-M) may* also undergo the loss of heterozygosity (LOH) or mutations, particularly in metastatic tumors [53]. As revealed by a pan-cancer study, *MHC* LOH occurs in 17% of various cancers and is a predictive biomarker for poor responses to immunotherapy in NSCLC [54]. Although rare, complete down-regulation of surface MHC-I expression due to loss-of-function mutations/deletions in *β2-M* or the *MHC-I* heavy chain is irreversible.

*Down-regulation of APM by Epigenetic Silencing.* The promoter hypermethylation of APM genes, such as *MHC*, *β2-M* and transporter of antigenic peptide, frequently gives rise to transcriptional silencing. The up-regulation of MHC-I expression is attributed to the demethylation of *MHC* genes [55]. This may also be triggered by the sensing of cytosolic double-stranded ribonucleic acid (RNA) (dsRNA) derived from endogenously demethylated endogenous retrovirus (*ERV*) genes [56]. ERVs as remnants of ancient viral infections account for approximately 8% of the human genome [57]. Instead of being normally silenced via hypermethylation, they can be reactivated by DNA methyltransferase inhibitors, which thereby induce the dsRNA sensing pathway [56]. The pathway activates interferon type I (IFN-I) cellular response and the up-regulation of MHC-I expression mediated by nuclear factor kappa-light-chain-enhancer of activated B cells (NF-κB). In addition, histone modifications like H3K27me3 and histone deacetylation have been demonstrated to silence APM genes in tumor cells [76].

*Dysregulation of MHC-I transcription.* Transcriptomic regulation of MHC-I is tightly controlled to elicit an appropriate immune response. Interferon-gamma (IFN-γ), a multifunctional cytokine secreted by activated immune cells in the inflamed tumor microenvironment, serves as a key regulator of MHC-I expression (Fig. 3B) [58]. IFN-γ regulates the expression of *MHC* via the Janus kinase 1/2 (JAK1/2)-signal transducer and activator of transcription 1 (STAT1) axis and induces the expression of NLR Family CARD Domain Containing 5 (NLRC5) [51]. NLRC5 as a trans-activator, plays a leading role in regulating the expression of MHC-I. It creates a scaffold with regulatory factor X (RFX), cAMP-response element binding protein (CREB), activating transcription factor 1 (ATF1), nuclear factor-Y (NF-Y) and other regulatory DNA-binding proteins in proximal *MHC* gene promoters, and constitutes the class I trans-activator (CITA) complex [59]. These transcription factors and regulatory complexes are also found in the promoters of *MHC*, *β2-M* and other APM genes. Deleting *NLRC5* in mice in vivo results in loss of MHC-I expression but does not affect MHC-II expression. This confirms that NLRC5 plays a specific regulatory role in the transcription of MHC-I [60]. This pathway also induces the expression of interferon regulatory factor 1/2 (IRF1/2), which binds to interferon-stimulated response elements (ISREs) in proximal *MHC* gene promoters [61]. In NSCLC, inactivating mutations in *JAK1*, *JAK2* or *STAT1* are frequently observed. These mutations impair the phosphorylation of STAT1, disrupt the regulation of NLRC5 and IRF1, dramatically reduce the expression of *MHC-I*, and promote resistance to ICIs [62]. In addition, tumor cells can evade immune surveillance through epigenetic silencing mediated by the polycomb repressive complex 2 (PRC2), suppression of IFN-γ signaling by double homeobox protein 5 (DUX5), and loss of NLRC5 expression [51].

*Post-transcriptional and post-translational regulation.* Non-coding RNAs like microRNA-125a (miR-125a) and long non-coding RNA (lncRNA) LINK-A can directly suppress the translation or promote the degradation of *MHC-I* or transporter 2, ATP binding cassette subfamily B member (TAP2) transcripts [63, 64]. These regulatory mechanisms have been linked to poor ICI efficacy in certain solid tumors like triple-negative breast cancer (TNBC). In addition, post-translational modifications also contribute to the down-regulation of MHC-I. These include the ER-associated degradation (ERAD) of misfolded MHC-I, the myelin and lymphocyte protein 2 (MAL2)-mediated endocytosis of MHC-I and autophagy lysosome dependent clearance [65]. Although direct evidence in lung cancer is limited, the highly active autophagy noted in this context suggests similar mechanisms may be involved.

#### Dysfunction of the cGAS-STING pathway

Emerging evidence highlights that innate immune sensing and genomic instability play a vital role in shaping immunotherapy resistance in NSCLC. STING is an adaptor protein located in the ER, while cGAS works as a cytosolic sensor detecting aberrant dsDNA [66]. In NSCLC cells, cytosolic dsDNA can originate from sources such as genomic instability, mitochondrial DNA leakage or viral infection. Upon recognition, activated cGAS catalyzes the generation of cyclic GMP-AMP (cGAMP) binding to and activating STING [67, 68]. This activation initiates a downstream signaling cascade involving TANK-binding kinase 1 (TBK1) and inhibitor of NF-κB (IκB) kinase (IKK), which leads to the phosphorylation and nuclear translocation of interferon regulatory factor 3 (IRF3) and NF-κB. These transcription factors induce IFN-I and proinflammatory cytokine production, and promote innate and adaptive anti-tumor immunity [68, 69].

**Fig. 4:**
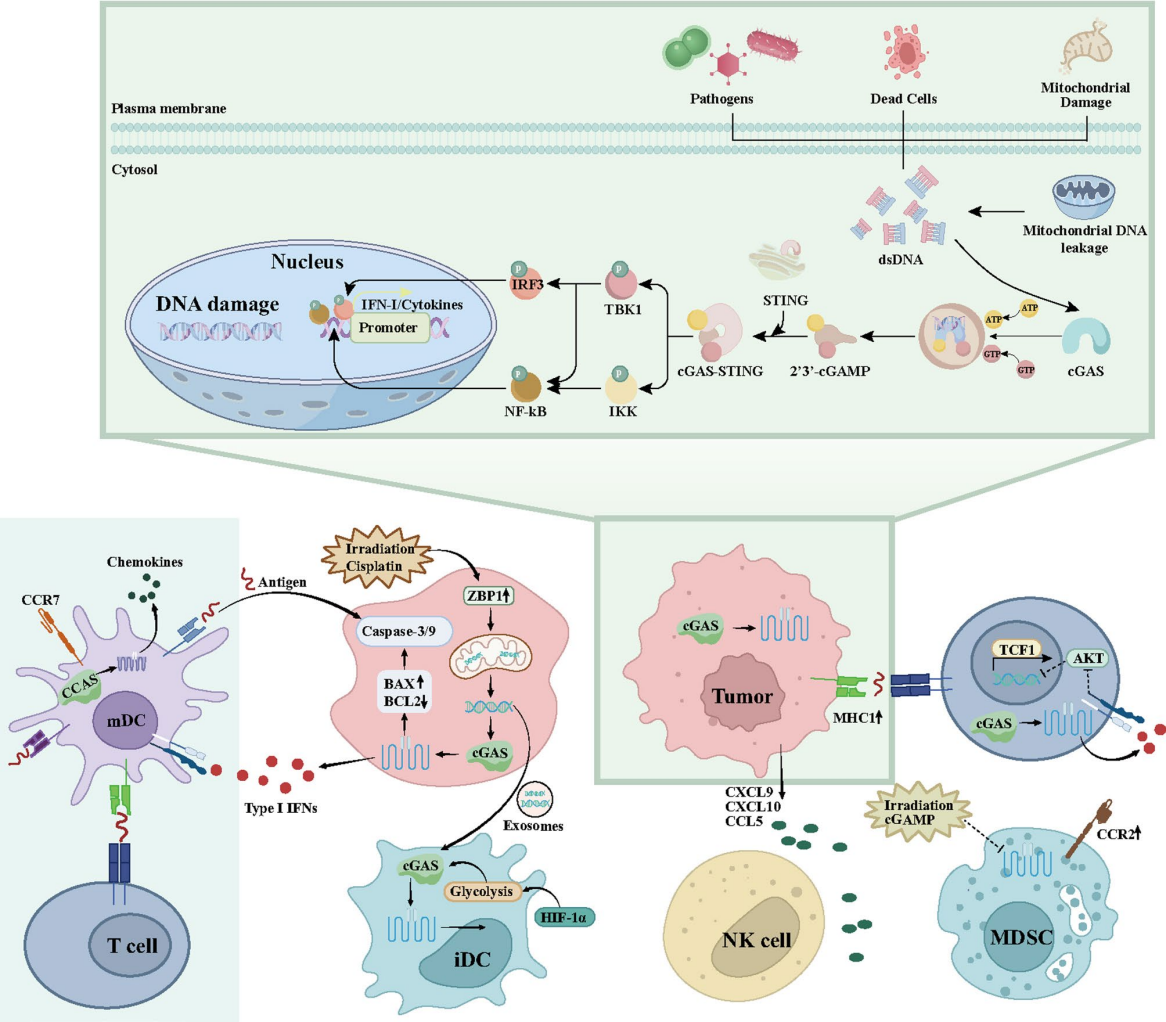
Tumor Suppression Mechanism of cGAS-STING. The cGAS-STING tumor suppression pathway initiates when cytosolic DNA activates cGAS, triggering 2’3’-cGAMP synthesis. cGAMP binding to STING activates transcription factors IRF3 and NF-κB, inducing expression of immune genes (*e.g*., IFN-I, cytokines). This intricate cascade recruits and activates immune cells to recognize and eliminate tumor cells, highlighting its therapeutic potential

Beyond its classical antiviral role, cGAS-STING signaling is of importance to reshape the tumor immune microenvironment (Fig. 4) [70]. In DCs, tumor-derived DNA can be internalized via exosomes, which activate cGAS-STING and promote the production of IFN-I, the maturation of DCs, the up-regulation of MHC-I and the suppression of lysosomal degradation of tumor antigens [71, 72]. These enhance antigen presentation and subsequent CD8⁺ T cell activation in tumor-draining lymph nodes [72, 73]. Moreover, glycolytic reprogramming mediated by hypoxia-inducible factor 1 alpha (HIF-1α) in DCs further amplifies STING activity in NSCLC, which forms a positive feedback loop [73]. In addition, cGAS-STING activation also promotes the secretion of chemokines (e.g., C-X-C motif chemokine 9 (CXCL9), CXCL10 and C-C motif chemokine ligand 5 (CCL5)), and enables CTL infiltration and tumor recognition. Recent studies have underscored that cGAS-STING activation is clinically significant in NSCLC, and have revealed an association between the enhanced expression of cGAS, CCL5 and CXCL10 and a good prognosis in NSCLC patients receiving chemotherapy and immunotherapy [74]. Nevertheless, lung cancer also evades immune surveillance by inhibiting cGAS-STING via multiple pathways, including glycolysis-dependent activation of NOP2/Sun RNA methyltransferase 2 (NSUN2). Glucose-bound NSUN2 stabilizes three prime repair exonuclease 2 (TREX2), limiting the accumulation of cytosolic dsDNA and suppressing cGAS/STING signaling. This cascade reduces CD8^+^ T cell infiltration, promoting tumorigenesis and resistance to anti-PD-L1 immunotherapy [75]. The decreased expression of cGAS and STING in the peripheral blood CD8^+^ T cells of cancer patients underscores that these molecules play a critical role in the function of CD8^+^ T cells [76]. Furthermore, STING activation enhances the recruitment of natural killer (NK) cells through IFN-I and chemokine secretion. Activated NK cells exert cytotoxicity in a bystander fashion, particularly against tumors showing resistance to killing mediated by T cells [77]. Notably, MET-amplified lung cancer resists immunotherapy. Studies show this correlates with impaired STING signaling, reducing CD8^+^ T and NK cell infiltration while increasing exhaustion markers in both cell types. This collectively impairs anti-tumor immunity [78]. STING signaling plays a dual role in modulating MDSCs. In most contexts, it suppresses the accumulation and function of MDSCs, which thereby alleviating immunosuppression [79]. However, some evidence, particularly in NSCLC and irradiated tumor models, suggests that STING activation may promote the recruitment of C-C chemokine receptor 2 (CCR2) -MDSCs, which possibly exacerbates immune tolerance after RT or STING agonist treatment [80]. Taken together, these findings underscore that the loss or suppression of cGAS-STING signaling represents a key resistance mechanism in NSCLC.

### Tumor-Extrinsic mechanisms

#### Immunosuppressive TME

TME is a highly dynamic and immunosuppressive ecosystem that comprises tumor, stromal and immune cells, extracellular matrix, blood vessels and various soluble factors [81]. The composition and spatial organization of TME have a profound influence on immune evasion, tumor progression and immunotherapy responses. Multiple immunosuppressive cell types, including tumor-associated macrophages (TAMs), MDSCs and tumor-associated neutrophils (TANs), actively inhibit antitumor immunity mediated by CD8⁺ T cells through direct contact or secretion of suppressive cytokines (e.g., IL-10 and TGF-β), reactive oxygen/nitrogen species and immune checkpoint ligands (Fig. 2C).

Mounting evidences have shown that the immunosuppressive rewiring of the TME is largely driven by tumor-intrinsic metabolic reprogramming [82]. The shift of tumor cells toward aerobic glycolysis (Warburg effect), lipid accumulation and enhanced amino acid uptake (e.g., glutamine) supports uncontrolled proliferation and survival [83]. These metabolic alterations lead to the depletion of nutrients, the accumulation of immunosuppressive byproducts and the establishment of a metabolically hostile environment. For instance, excess glucose consumption by tumor cells reduces its availability to immune cells, while high lactate levels acidify the TME and impair the functions of effector T cells, NK cells and DCs. Lactate also promotes the differentiation and suppressive activity of Tregs, and skews TAMs toward an M2-like phenotype fostering immune tolerance [84].

Tumor-derived metabolites such as lactic acid, prostaglandin E2 (PGE2), fatty acids (FAs), cholesterol, D-2-hydroxyglutarate (2-HG), kynurenine (KYN) and adenosine (ADO) may disrupt the metabolism and function of CD8⁺ T cells [85]. These metabolites mediate intercellular metabolic competition between tumor and immune cells as well, which causes nutrient starvation, hypoxia and oxidative stress. PGE2, for example, facilitates the expansion of MDSCs, differentiation of Tregs and suppression of type 1 conventional DCs (cDC1) via the cAMP–protein kinase (PKA) pathway, while reducing the production of IL-2 and IFN-γ by effector T cells [86]. Long-chain FAs (LCFAs) and lipid accumulation in CD8⁺ T cells induce mitochondrial dysfunction and oxidative stress, promoting the exhaustion of T cells [87]. Cholesterol accumulation increases the expression of checkpoint molecules (PD-1 and TIM-3), while KYN acts via the aryl hydrocarbon receptor (AhR) to inhibit the maturation of DCs and the cytotoxicity of CD8⁺ T cells. Similarly, 2-HG impairs the production of chemokines and recruits immunosuppressive macrophages, which contributes to the exclusion of T cells [88].

Meanwhile, MDSCs in the TME undergo their own metabolic rewiring and favor glycolysis and glutaminolysis. MDSC-derived ROS, nitric oxide (via inducible nitric oxide synthase (iNOS)) and arginase-1 (Arg-1)-mediated arginine depletion collectively suppress the proliferation of T cells and the expression of CD3 [89]. In NSCLC, increased circulating or tumor-infiltrating MDSCs are related to poor CD8⁺ T cell responses and reduced effectiveness of PD-1 blockade. Likewise, TANs recruited by tumor-derived chemokines can switch from anti-tumor to pro-tumor phenotypes under TME-derived cues [90]. Pro-tumor TANs release IL-10 and TGF-β, form neutrophil extracellular traps (NETs) and produce arginase and ROS, which thereby impede the trafficking and activity of T cells [91].

Beyond cellular elements, the structural and physiological features of the TME, such as abnormal vasculature and localized hypoxia, further restrict the infiltration of immune cells. Hypoxia promotes the expression of PD-L1 and other immunosuppressive molecules and disrupts mitochondrial function in T cells through the inhibition of HIF-1α signaling and oxidative phosphorylation, which contributes to immune exhaustion [92]. Under glucose- and oxygen-limited conditions, tumor-infiltrating T cells exhibit reduced mitochondrial mass, increased ROS burden and diminished glycolytic and oxidative capacities. This loss of metabolic fitness undermines their persistence and effector function.

Overall, tumor-intrinsic metabolic rewiring generates an immunosuppressive TME that suppresses antitumor immune cells through metabolic competition, nutrient deprivation and toxic byproduct accumulation. The understanding of the bidirectional crosstalk between tumors and immune cell metabolism not only reveals key vulnerabilities but also opens up new avenues for treatment interventions.

#### Exhaustion of CD8^+^ T cells

CD8^+^ T cells are quite instrumental in combating cancer. Tumors responding well to ICI therapy usually exhibit higher levels of CD8^+^ T cell infiltration and rapid and extensive cell proliferation in the bloodstream [93, 94]. By comparison, “cold” tumors, which have the minimal infiltration of CD8^+^ T cells, typically show poor or no response to immunotherapy [95]. CD8^+^ T cells undergo a developmental process, transition from naive to effector cells, and further differentiate into memory cells [96]. Nevertheless, prolonged antigen exposure can lead to the exhaustion of T cells, which is a state characterized by reduced functionality [97]. The severe exhaustion of T cells is a critical challenge in effective tumor immunotherapy. When T cells are exposed to tumor antigens for an extended period, continuous antigen signaling causes a decline in the activity of T cells, which eventually results in exhaustion. Exhausted T cells lose their potent response to antigens [98]. For one thing, inhibitory receptors, including PD-1, CTLA-4, TIM-3, etc., demonstrate an increase in expression level during the exhaustion of T cells. These receptors negatively regulate the activation and function of T cells and stop T cells from mounting effective antitumor responses. For another, exhausted T cells gradually lose the ability to secrete key immune-activating cytokines like TNF-α and IFN-γ. More than that, T-cell metabolic functions progressively decline under prolonged antigen exposure and chronic inflammatory conditions. This leads to energy depletion, which obstructs the maintenance of normal immune function. CD8^+^ T cells are main CTLs that mediate antitumor immunity through the recognition of MHC-I molecules on tumor cell surfaces (Fig. 2D). Exhausted CD8^+^ T cells exhibit the diminished capability of antigen clearance, the reduced secretion of cytokines (like IFN-γ) and the overexpression of inhibitory receptors (like PD-1 and TIM-3). This functional decline keeps the immune system from effectively targeting and eliminating tumors, which contributes to reduced immunotherapy efficacy or disease progression. Zhang et al. used single-cell sequencing technology for comprehensively characterizing T cells in NSCLC. They found that the abundance of exhausted T cells and Tregs was linked to poor patient prognosis. Beyond that, exhausted T cells were dominant in tumor tissues, and their gene expression profiles indicated functional impairments like reduced cytotoxicity and diminished proliferative capacity. This suggests that exhausted T cells are likely to promote tumor immune evasion, which leads to immunotherapy resistance in lung cancer [99]. To conclude, exhausted T cells, including impaired tumor cell clearance, facilitate the immune evasion and progression of tumors, which results in resistance to ICI therapies [100].

#### Gut and lung microbiomes

Gut and lung microbiomes, critical regulators of host immunity, play pivotal roles in modulating antitumor immune responses, predicting immunotherapy efficacy and elucidating therapeutic resistance mechanisms [101]. These microbial communities influence the tumor immune microenvironment, remodel host metabolic states and interfere with antigen presentation, thereby shaping the responses to ICIs and facilitating immune escape. Increasing clinical evidences have shown that the composition of the gut microbiota is closely associated with ICI outcomes. In particular, gut dysbiosis, typified by reduced microbial diversity, loss of beneficial taxa and pathogenic overgrowth, is commonly seen in non-responders. Conversely, responders exhibit the enrichment of *Akkermansia muciniphila*, which stimulates the production of IL-12 by DCs, enhances the activation of CD8⁺ T cells and thereby improves anti-PD-1 therapy responses [102, 103].

Whether it is caused by underlying disease or antibiotic administration, the dysbiosis of the gut microbiota may have a negative impact on the response to immunotherapy [104, 105]. Antibiotics exert a dual impact on cancer immunotherapy outcomes [106]. On the one hand, gut microbiome disruption may hinder the effectiveness of ICIs by interfering with pattern recognition receptor (PRR) signaling and weakening local and systemic immune responses. Furthermore, dysbiosis may lead to the selective enrichment of pathogenic and drug-resistant microbes. Such microbes include *Candida species*, *Clostridium difficile*,* Carbapenem-resistant Enterobacteriaceae*, *Vancomycin-resistant enterococcus (VRE) and Extended-spectrum β-lactamase-producing bacteria (ESBL)*. They are known to compromise therapeutic efficacy and patients’ survival. On the other hand, antibiotics may selectively eliminate pro-tumor microbial populations and partially restore mucosal immune responsiveness. Studies have shown that the replenishment of beneficial commensals enhances ICI efficacy and reduces tumor burden. For instance, the oral administration of *Bacteroides fragilis* in melanoma-bearing mice improved the efficacy of CTLA-4 blockade by promoting antigen-specific T cell responses [107]. Similarly, the oral supplementation with probiotic *Bifidobacterium* restored the sensitivity to anti-PD-L1 therapy by enhancing the activity of antitumor CD8⁺ cytotoxic T cells and inducing the maturation of DCs in a murine melanoma model [108]. Routy et al. analyzed gut microbiomes in 249 patients receiving PD-1 immunotherapy for urothelial carcinoma, NSCLC, or renal cell carcinoma. Among them, 69 patients given antibiotics (for dental, urinary, or respiratory infections) showed significantly reduced OS and accelerated disease progression *versus* unexposed patients, suggesting antibiotic-induced dysbiosis compromises ICI efficacy [103].

Importantly, microbial metabolism exhibits a strong association with immune resistance. This is especially evident in tryptophan pathway, which critically regulates T-cell functionality. In ICI-responsive NSCLC patients, the increased activity of the tryptophan pathway, especially through tryptophanyl-tRNA synthetase (WARS)-mediated tryptophanyl-lysine modification, facilitates hormone receptor interactor 12 (TRIP12)-driven degradation of nuclear factor of activated T-cells, cytoplasmic 1 (NFATc1), a PD-1 transcription factor. This leads to the down-regulation of PD-1 expression and enhanced T cell activation [109, 110]. Conversely, an elevated kynurenine/tryptophan (Kyn/Trp) ratio indicates the heightened activity of indoleamine 2,3-dioxygenase 1 (IDO1) in non-responders. This results in tryptophan depletion, T cell suppression and Treg differentiation, which promotes immune tolerance. Moreover, ICI-resistant patients accumulate 3-hydroxyanthranilic acid (3-HAA), a tryptophan metabolite. This compound drives conventional DC expansion and TGF-β secretion, thereby exacerbating immunosuppression and resistance [111, 112]. Consequently, co-expression of IDO1 and PD-L1, elevated Kyn/Trp ratio, and abnormal 3-HAA accumulation have been proposed as metabolic hallmarks of gut microbiota-mediated ICI resistance, offering a theoretical rationale for combined IDO and PD-1 blockade strategies.

Although research on the lung microbiome has lagged, emerging evidence suggests its distinctive role in the modulation of local immune escape and therapeutic resistance in NSCLC [113]. For instance, *Streptococcus* and *Veillonella* are enriched in lung tumors and associated with the activation of the T helper cell 17 (Th17) pathway, driving chronic inflammation, and fostering immunosuppressive microenvironments [114]. These organisms may impair immune surveillance by activating γδ T cells, inhibiting the maturation of APCs and reducing the infiltration of CD8⁺ T cells. It has also been demonstrated that disrupting the pulmonary microbiota promotes Treg and M2 macrophage accumulation, fostering a highly immunosuppressive TME and upregulating immune checkpoints (e.g., PD-L1). This cascade ultimately impairs T-cell function [115]. Apart from that, disturbances to the lung microbiome, such as antibiotic use or pulmonary infections, significantly impair ICI efficacy, underscoring the bidirectional crosstalk between lung microbes and antitumor immunity [113].

## New strategies to overcome immunotherapy resistance

### Combination of chemotherapy and RT

**Fig. 5:**
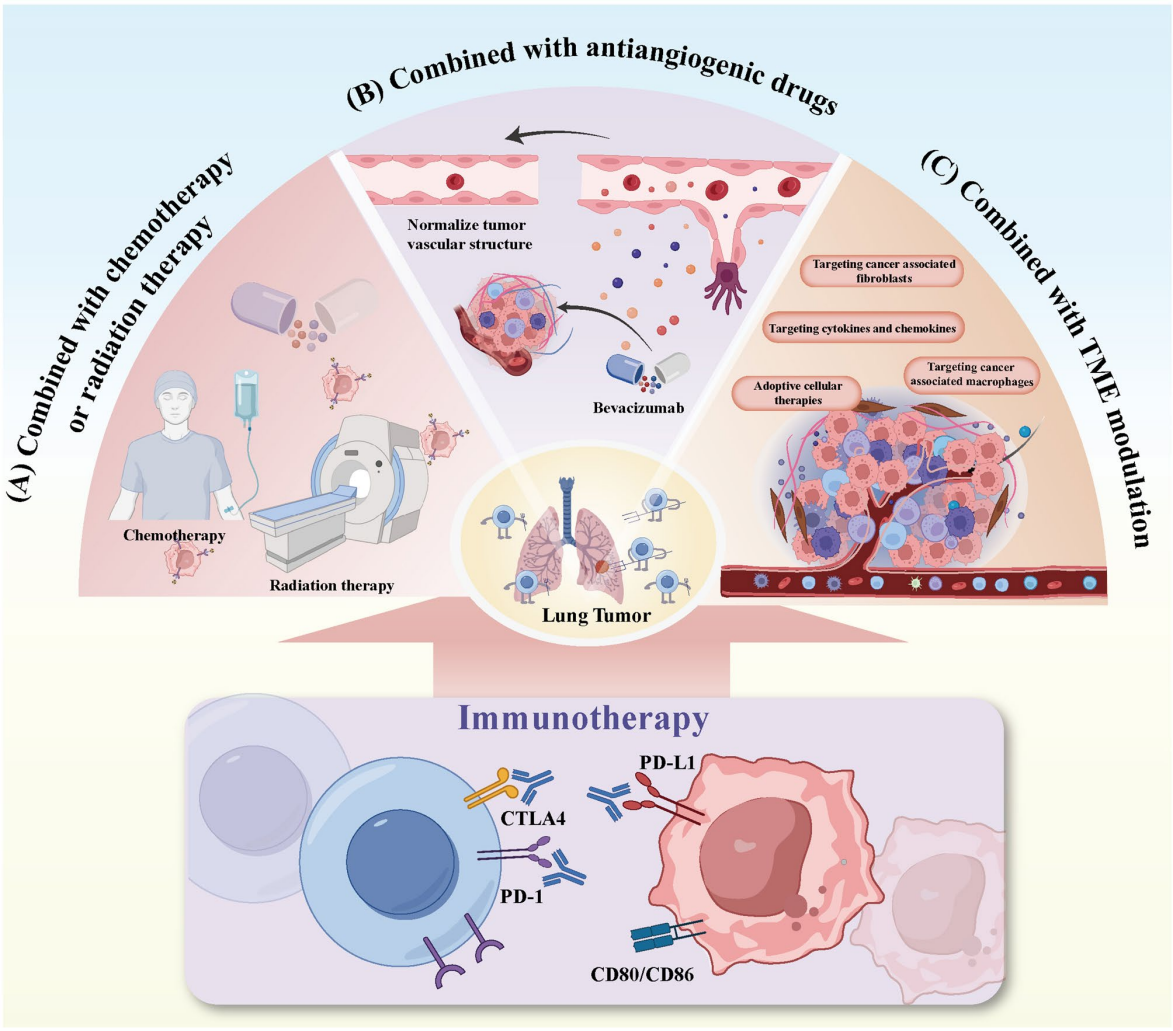
New Treatment Strategies to Overcome Immunotherapy Resistance. (**A**) Combining chemotherapy or RT with immunotherapy can lead to tumor cell death. This causes the release of more TAAs, which thereby activate anti-tumor immune response. (**B**) Combined treatment with ICIs and antiangiogenic drugs. Anti-angiogenic drugs can target signaling factors like VEGF, which inhibits their overexpression, reduces vascular permeability, and improves tumor perfusion and blood flow to temporarily normalize the tumor vascular system. (**C**) Combination strategies can be adopted to mobilize the cellular and molecular components of TME involved in anti-tumor immunity in various stages, convert “cold tumors” into “hot” ones, and provide new treatment options after the failure of immunotherapy

Combining chemotherapy or RT with immunotherapy has become a useful strategy to overcome resistance to cancer immunotherapy. Chemotherapy uses chemical agents for killing or inhibiting cancer cell growth and division, whereas RT employs high-energy radiation to directly destroy tumor cells or halt their growth. When used in combination with immunotherapy, both chemotherapy and RT can address resistance to immunotherapy through multiple primary mechanisms (Fig. 5A). On the one hand, chemotherapy and RT can induce the death of tumor cells, which leads to the release of tumor-associated antigens (TAAs). These TAAs can be recognized by the immune system, which triggers an antitumor immune response. The release of antigens enhances the ability of the immune system to detect and attack cancer cells in a more effective way. On the other hand, chemotherapy and RT can reduce the number of immunosuppressive cells like Tregs and MDSCs in the TME. By depleting these cells, the immune system can mount a stronger response, which increases the efficacy of immunotherapies like checkpoint inhibitors [116]. In both solid tumors and lymphomas, the combination of PD-1 inhibitors and chemotherapy has shown promising results. For instance, Falvo et al. [117] explored the use of chemotherapeutic agents, cyclophosphamide and vinorelbine, in the treatment of TNBC. These findings indicate that these drugs could activate APCs, suppress the growth of both local and metastatic tumors through T-cell-mediated mechanisms, and thereby enhance the efficacy of anti-PD-1 therapy. In like manner, Wang et al. [116] demonstrated the effectiveness of combining decitabine with camrelizumab (a PD-1 inhibitor) in Hodgkin lymphoma patients who previously failed treatment with PD-1 inhibitors. This combination greatly improved objective response rate (ORR) and PFS, with 52% of patients achieving an objective response, including 36% who experienced complete remission.

Building on this synergy, increasing evidence has highlighted the critical role of DNA damage response (DDR) pathways in further shaping the immunologic effects of chemotherapy and RT. DDR is activated upon DNA damage and comprises multiple processes, including damage sensing, the activation of cell cycle checkpoints, DNA repair and the induction of cell death when damage is irreparable. Beyond these cellular effects, DNA damage has been observed to facilitate protective immune cell infiltration while simultaneously upregulating immunosuppressive molecules like PD-L1 [118, 119]. An increasing number of preclinical and clinical studies show that changes in tumor DDR pathways are closely related to sensitivity to ICIs [120, 121]. As a consequence, DDR inhibitors combined with RT and immunotherapy appear to be a promising strategy for achieving synergistic therapeutic effects and improving outcomes across multiple tumor types. Mechanistically, DDR deficiency augments ICI efficacy via immune remodeling [122]. First, defective DNA repair elevates somatic mutations in exomes, increasing neoantigen-driven CD8⁺ T cell infiltration and cytotoxic activity [123, 124]. Second, unrepaired DNA fragments may accumulate in the cytoplasm, which triggers the cGAS-STING-TBK1-IRF3 cascade. This leads to IFN-I production and initiates innate immune responses. For example, tumors harboring mutations in key DDR genes like breast cancer susceptibility protein 1 (*BRCA1*) and ataxia telangiectasia mutated (*ATM*) often display elevated levels of cytosolic DNAs. This enhances the activation of the STING pathway and increases tumor immunogenicity, which contributes to a more durable response to ICIs [125]. On balance, DDR inhibitors such as those targeting poly ADP-ribose polymerase (PARP), DNA-dependent protein kinase (DNA-PK) and ATM/ataxia telangiectasia and Rad3-related protein (ATR) not only sensitize tumors to RT, but also reprogram the immune landscape. When used in combination, RT, DDR inhibition and immunotherapy form a synergistic triad enhancing antitumor efficacy and possibly overcome therapeutic resistance [126]. This integrated approach offers a compelling strategy to improve clinical outcomes and opens new avenues for precision oncology.

While combination therapies offer substantial promise, chemotherapy and RT can also induce toxicity in normal cells, which causes adverse side effects. Moreover, the complexity of the TME presents challenges because of different patient responses to combination treatments. Thus, personalized therapy will remain a crucial focus in the future to make sure that treatments are tailored to individual patients and their tumor characteristics.

### Combination with antiangiogenic agents

Angiogenesis means that new blood vessels are formed from pre-existing capillaries or post-capillary venules. Tumors promote angiogenesis to secure the oxygen and nutrients required for their growth and survival, which makes angiogenesis a critical process in tumor proliferation and persistence. In recent years, 11 anti-angiogenic agents have been approved either as monotherapies or in combination with other immunotherapies or targeted therapies for advanced cancer treatment [127]. Anti-angiogenic drugs target signaling molecules like VEGF, inhibit its overexpression, reduce vascular permeability, and improve tumor perfusion and blood flow, which thereby temporarily normalizes the tumor vasculature. However, these drugs can also trigger the excessive pruning of the tumor vasculature in a dose- and time-dependent manner, which can induce hypoxia and immune suppression, including the up-regulation of PD-L1 expression [128]. Immunotherapy, particularly ICIs, is complementary to antiangiogenic agents. On the one hand, anti-angiogenic drugs reverse VEGF-induced immunosuppression and normalize the tumor vasculature, which enhances the infiltration and perivascular accumulation of CD8^+^ CTLs expressing IFN-γ [127]. This can increase the activity of T cells against tumor-specific antigens (TSAs) and other immune effector cells. On the other hand, ICIs restore an immune-supportive environment through the activation of effector T cells and the up-regulation of IFN-γ secretion. This further normalizes the tumor vasculature, boosts T-cell infiltration and cytotoxic activity, and improves drug delivery (Fig. 5B) [129, 130]. This synergy allows for a reduction in ICI dosage, which lowers the risk of adverse events [131]. A study reported in 2018 assessed the combination of sintilimab, an anti-PD-1 antibody, with a multi-target anti-angiogenic tyrosine kinase inhibitor (anlotinib) as a first-line therapy for NSCLC patients. It showed promising results, with 72.7% of patients (16 out of 22) achieving partial remission and 15-month median PFS. Overall, the efficacy of combining immunotherapy with anti-angiogenic agents in advanced NSCLC patients was demonstrated [132]. Future research should pay attention to the optimization of combination therapy regimens and the exploration of the best drug combinations and dosing strategies to further improve patients’ survival rates while minimizing adverse effects.

### TME modulation

The TME is a dynamic and complex network where all kinds of immunosuppressive cells, secreted molecules and signaling pathways are active in contributing to immune tolerance and tumor progression [91]. Chronic inflammatory conditions may alter or skew the differentiation of immune cells in lung cancer, which weakens anti-tumor responses and leads to resistance against ICIs [133]. Cytokines within the TME mediate communication among immune cells and play an indispensable role in cancer progression in both bloodborne and solid tumors (Fig. 5C). To date, IL-2 and IFN-α are the only cytokines approved as standalone therapies for melanoma and renal cell carcinoma [134]. Research on NSCLC remains limited, although multiple cytokines are being investigated in combination with immunotherapies, particularly PD-1/PD-L1 inhibitors, for a variety of solid tumors [91]. IL-15, which is a key cytokine involved in the development, proliferation and effector functions of NK cells and T lymphocytes, has demonstrated anti-tumor activity in preclinical studies. An early-phase clinical trial recently evaluated the safety of IL-15 in advanced solid tumors, including NSCLC. It revealed that the subcutaneous administration of IL-15 at a dosage of 2 µg/kg/day was safe and effective [135]. A separate phase I trial tested the combination of the IL-15 superagonist ALT-803 with nivolumab in patients who suffered from previously treated stage IIIB or IV NSCLC. This combination was well tolerated by a majority of patients, with a median OS of 17.4 months. Some patients showed signs of disease stabilization or partial response. Treatment increases the number of circulating NK and CD8^+^ T cells to a great degree, which indicates that the combination of ALT-803 and nivolumab holds promise as a novel immunotherapeutic strategy [136].

Adoptive cellular therapy (ACT), which involves the ex vivo expansion and modification of patients’ own immune cells followed by reinfusion into target tumors, has gained traction as an immunotherapy approach [137, 138]. The common forms of ACT include T-cell receptor (TCR) gene, tumor-infiltrating lymphocytes (TILs) and chimeric antigen receptor (CAR) T-cell therapies [139]. Recent studies that combine ACT with ICIs have demonstrated significant improvements in the modification of the TME and the enhancement of anti-tumor effects. A phase I clinical trial showed that 20 advanced NSCLC patients progressing on nivolumab (anti-PD-1) monotherapy received autologous TIL therapy followed by continued nivolumab treatment. A total of 11 patients achieved disease stabilization or partial response, and two achieved complete remission after 1.5 years of continuous therapy. These findings suggest that TIL therapy could represent a promising strategy for treating metastatic lung cancer resistant to PD-1 [140]. On the whole, the complexity and dynamic nature of the TME have profound implications for the efficacy of immunotherapy. Combination strategies engaging multiple aspects of anti-tumor immunity may be beneficial to converting “cold tumors” into “hot” ones, which provides new directions for treatment options after immunotherapy failure.

### Epigenetic modulation to restore immunogenicity

DNA methylation, chromatin remodeling, histone acetylation and other epigenetic modifications play an important part in the regulation of gene expression without changing DNA sequences. In cancer, epigenetic dysregulation silences TAAs, impairs antigen presentation, reduces immune cell trafficking and promotes an immunosuppressive TME [141], ultimately enabling immune evasion. Epigenetic agents such as histone deacetylase inhibitors (HDACis), DNA methyltransferase inhibitors (DNMTis) and bromodomain and extraterminal domain inhibitors (BETis) are designed to reverse these suppressive programs and restore tumor immunogenicity.

**Fig. 6:**
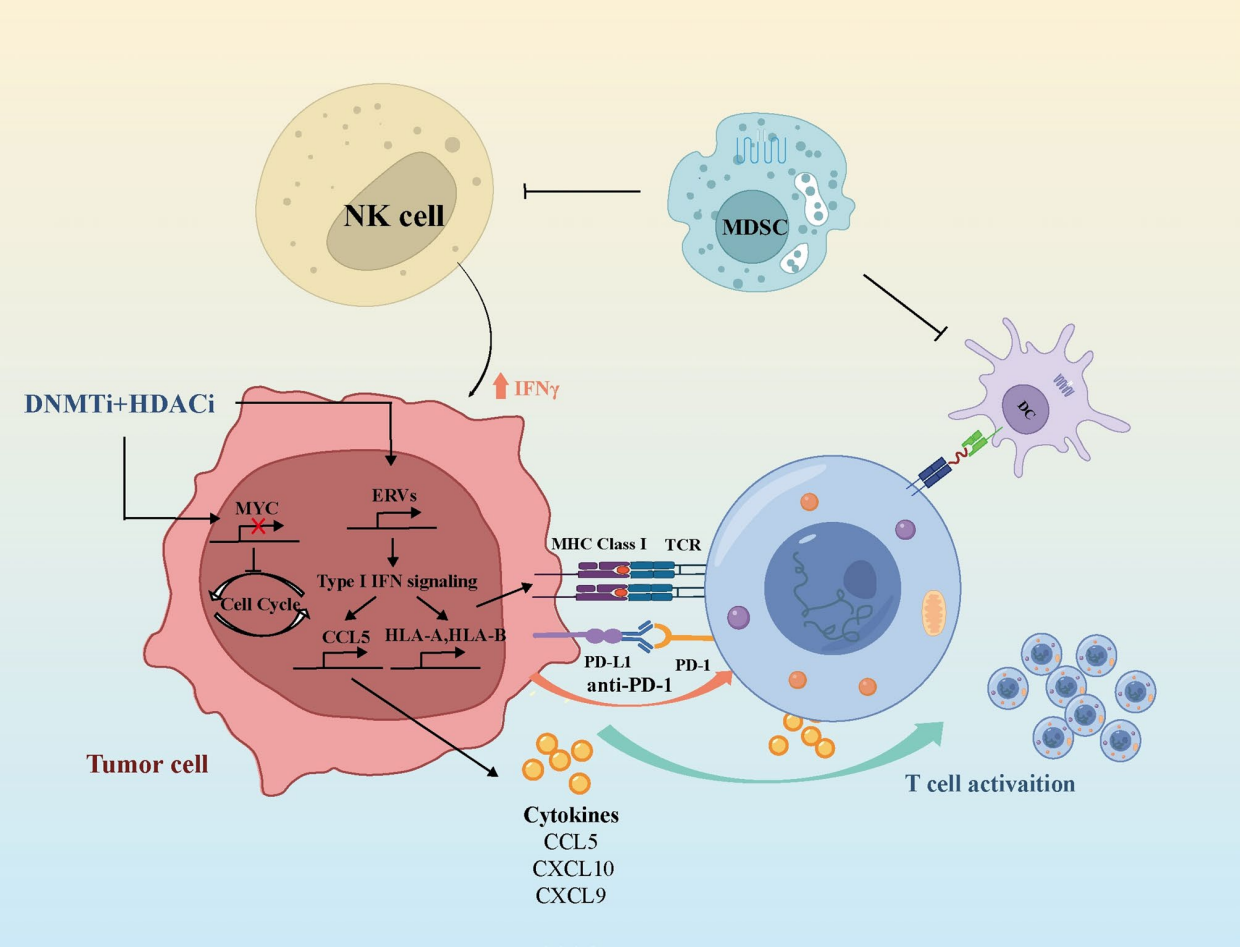
Action Mechanisms of Anti-PD-1/Anti-PD-L1 Immunotherapy Combined with HDACi and DNMTi. DNMTis and HDACis cooperatively enhance anti-tumor immunity through distinct but complementary mechanisms. DNMTis demethylate endogenous dsRNAs, including ERVs, activating TLR3/MDA5 sensors to trigger Type I/III interferon signaling. This cascade upregulates APM and MHC-I surface expression. Concurrently, DNMTis demethylate the promoter regions to induce the expression of CCL5 and cancer-testis antigens. HDACis similarly boost antigen presentation and increase PD-L1 expression. Both agents promote T-cell activation against tumors and suppress MDSCs, collectively fostering an immunogenic TME

HDACis act by preventing the removal of acetyl groups from histones, which maintains an open chromatin structure, facilitating the transcription of immune-related genes. In NSCLC, HDACis upregulate the expression of MHC class I and II molecules, TAAs and co-stimulatory ligands (e.g., CD80 and CD86), which thereby enhances the recognition of tumor cells by APCs and CTLs [142, 143]. Furthermore, HDACis stimulate the production of chemokines such as CCL5, CXCL9 and CXCL10, promoting the infiltration of CD8⁺ T cells into the TME. These immune-enhancing effects underlie the observed synergistic effects when HDACis are combined with ICIs targeting PD-1/PD-L1, leading to improved tumor control in preclinical models [144, 145]. DNMTis exert immunomodulatory effects via the reactivation of silenced genomic regions, notably endogenous retroviruses (ERVs), which are generally suppressed by DNA methylation. They increase the transcription of ERVs and reduce their methylation, which leads to the accumulation of dsRNA [146]. These dsRNA species activate pattern recognition receptors (PRRs) like Toll-like receptor 3 (TLR3) and melanoma differentiation-associated protein 5 (MDA5), triggering IFN-I signaling and enhancing antigen processing, MHC expression and CCL5 up-regulation [147, 148]. The immune-activating effects of DNMTis can be further amplified by their combination with HDACis [149]. By contrast, BETis function by blocking the activity of BET family proteins (e.g., bromodomain containing 2 (BRD2), BRD3 and BRD4). These BRDs “read” acetylated lysines on histones and facilitate the transcription of genes like myelocytomatosis oncogene (*MYC*) and B-cell lymphoma-2 (*BCL2*), promoting tumor growth and immune evasion. Among these proteins, BRD4 plays a central role in driving oncogenic transcriptional programs, particularly in *MYC*-driven cancers, including *KRAS*-mutant NSCLC. MYC is a nuclear phosphoprotein that not only participates in cell cycle progression, apoptosis regulation, and cellular transformation, but also suppresses IFN-I signaling. Impaired IFN- I signaling further hinders antigen presentation through downregulated MHC expression [150]. BETis downregulate these immunosuppressive genes, which thereby promotes antigen presentation, enhances DC activation and restores CTL-mediated cytotoxicity. Notably, BET inhibition also attenuates the recruitment of Tregs and MDSCs, contributing to a more immunostimulatory TME [151]. In murine lung cancer models, the triple combinations of HDACi, BETis and anti-PD-1 therapy largely prolonged survival and led to durable tumor regression in comparison with monotherapy arms [152, 153]. Michael et al. [143] demonstrated that the combination of DNMTis and HDACis holds great promise for enhancing cancer immunotherapy. On the one hand, this combinatorial treatment activates a transcriptional program centered on interferon-α/β, accompanied by the upregulation of antigen presentation machinery, partially mediated through the induction of dsRNA. On the other hand, the strategy suppresses MYC signaling and increases the expression of the T cell chemoattractant CCL5. In mouse models of NSCLC, this dual epigenetic therapy reverses tumor immune evasion and reprograms T cells from an exhausted state toward memory and effector phenotypes, thereby enhancing antitumor immune responses (Fig. 6). These findings highlight the potential of epigenetic agents as immune-sensitizing adjuvants and critical tools to reverse intrinsic or acquired resistance to ICIs.

These mechanistic insights have begun to be validated by several clinical trials. In the trial NCT02664181, NSCLC patients were treated orally with tetrahydrouridine (THU)-stabilized decitabine, a DNMTi, combined with nivolumab. This triple regimen showed a higher ORR compared with nivolumab alone [154]. Similarly, the trial NCT02998567 tested guadecitabine, which is a next-generation DNMTi, with pembrolizumab in advanced solid tumors like NSCLC. The combination was well-tolerated and induced durable disease stabilization, even in patients who were previously refractory to ICIs [155]. Early-phase studies are also investigating ZEN-3694 [156], BMS-986,158 [157] and other BETis in conjunction with PD-1/PD-L1 blockade to assess potential synergy. In summary, HDACis, DNMTis, and BETis are mechanistically distinct but complementary epigenetic agents that remodel the tumor-immune microenvironment. Their coordinated effects on antigen expression, IFN signaling, and immune checkpoint regulation provide promising avenues to overcome ICI resistance. Advancing these therapies for lung cancer and other malignancies necessitates additional clinical validation and biomarker-driven combination strategies.

### Emerging small molecule agents in immunotherapy

In parallel, emerging small molecule drugs that target intracellular immune regulators or tumor metabolism offer additional therapeutic potential by overcoming resistance via alternative or complementary pathways. Small-molecule immunomodulators exhibit superior pharmacokinetic properties, including favorable oral bioavailability, prolonged half-life, enhanced tumor and tissue penetration and the ability to cross cell membranes to engage intracellular targets. Small-molecule immunomodulators are also cost-effective and easier to manufacture, which makes them attractive candidates for clinical application [158]. With the evolution of immunotherapy, small molecules are increasingly being recognized as valuable adjuncts to antibody-based treatments. They have the potential to overcome resistance, broaden antitumor efficacy and achieve safer and more effective combination therapies [159]. Recent advances in tumor immunology have fueled the development of small-molecule agents that are capable of activating or modulating immune responses by targeting immune checkpoints, cytokine signaling, metabolic pathways, oncogenic kinases and innate sensing mechanisms.

*Small Molecule Immunomodulators Targeting Innate Immunity.* Various target classes and key therapeutic targets in innate immunity have come into focus, especially proteins that inhibit the STING pathway in innate immune signaling. With the elucidation of the mechanism of STING action and the progress of translational medicine research, agonists targeting STING have emerged as a promising agent for the development of antitumor drugs. These agonists encompass various types, including vadimezan, cyclic dinucleotides and their derivatives. Preclinical studies have shown that concurrent administration of STING agonists with ICIs inhibits tumor growth and overcomes resistance to PD-1 therapy [160]. Several STING agonists combined with ICIs, such as MK-145,472 [161] and TAK-676 [162], have shown promising outcomes in clinical trials, including notable reductions in lesion volume and significant tumor regression. Additionally, ongoing research aims to evaluate the potential of ICIs combined with STING agonist derivatives, including ADU-S100, BI1387446, and E7766, for cancer treatment [160].

*Small Molecule Immunomodulators Targeting Adaptive Immunity.* Small molecules targeting the adaptive immune system aim to enhance the activation, proliferation and effector function of T cells. These agents primarily focus on modulating T cell responses and immune checkpoint pathways. Small molecule inhibitors of PD-1/PD-L1, such as A22 and 4-phenylindoline derivatives, have shown potent inhibitory activity in disrupting PD-1/PD-L1 interactions, thereby restoring effector T cell function [158]. Cytokines including IFNs, interleukins (e.g., IL-2, IL-10), and granulocyte-macrophage colony-stimulating factor (GM-CSF) play pivotal roles in stimulating CD4⁺ and CD8⁺ T cell proliferation, activating dendritic cells, and enhancing tumor antigen presentation [163]. Retinoic acid-related orphan receptor gamma T (RORγt) agonists can activate Th17 and CD8⁺ cytotoxic T cells, promote IL-17A secretion, and reduce the presence of Tregs and co-inhibitory molecules like PD-1, alleviating immune suppression within TME [163]. Small molecule inhibitors may block TGF-β, a key immunosuppressive factor, disrupting tumor-induced immune tolerance and enabling immune activation [164]. Finally, SH2-containing inositol-5’-phosphatase 1 (SHIP1) inhibitors can significantly enhance the responsiveness of T and NK cells and restore their effector functions. They represent a promising strategy to overcome immune suppression and strengthen antitumor immunity [164].

*Small Molecule Immunomodulators Targeting the TME.* Small molecules targeting immunometabolic pathways within the TME are designed to reverse immune suppression through the modulation of amino acid metabolism or purinergic signaling. IDO1 inhibitors block the catabolism of tryptophan into immunosuppressive kynurenine, which is a process driving the anergy and dysfunction of T cells in the TME. Notwithstanding the limited efficacy of single-agent IDO1 inhibitors in clinical trials, the development of dual-target inhibitors against IDO1/tryptophan 2,3-dioxygenase (TDO) or IDO1/IDO2 holds promise for overcoming compensatory metabolic escape mechanisms [158]. To restore L-arginine levels, arginase inhibitors are critical for the proliferation and cytotoxic activity of CD8⁺ T and NK cells. By inhibiting arginase overactivity in tumor cells and MDSCs, these agents help to re-establish an immunostimulatory environment [165]. CD39/CD73 inhibitors and A2A adenosine receptor antagonists counteract adenosine-mediated immune suppression by blocking adenosine signaling or its enzymatic production. Targeting the adenosine axis enhances the activity of T and NK cells, and may also synergize with CAR-T cell therapy to overcome deficits in antigen presentation and improve antitumor efficacy [166].

In addition, there are small-molecule agents that targeting PRRs, modulating oncogenic pathways, and inhibiting immunity-associated kinases. Despite recent progress in developing these immunomodulators, the inherent interconnectivity of therapeutic pathways poses challenges for small-molecule drug design. Nevertheless, ongoing efforts to advance small-molecule immunotherapies hold promise for opening new therapeutic avenues in cancer.

### Combination with Antibody-drug conjugates and bispecific antibodies

Antibody-drug conjugates (ADCs) and bispecific antibodies (bsAbs) integrate precise tumor-targeting capabilities with potent cytotoxic or immunomodulatory functions. They are two helpful strategies to strengthen the efficacy of immunotherapy. When combined with ICIs, these antibody-based therapies not only improve the visibility of tumor antigens but also remodel the immunosuppressive TME through all sorts of synergistic mechanisms. This section discusses the immunological mechanisms and clinical progress of ADCs and bsAbs in combination therapy in a systematic way, and introduces the future directions of immunotherapy strategies based on ADCs and bsAbs.

**Fig. 7:**
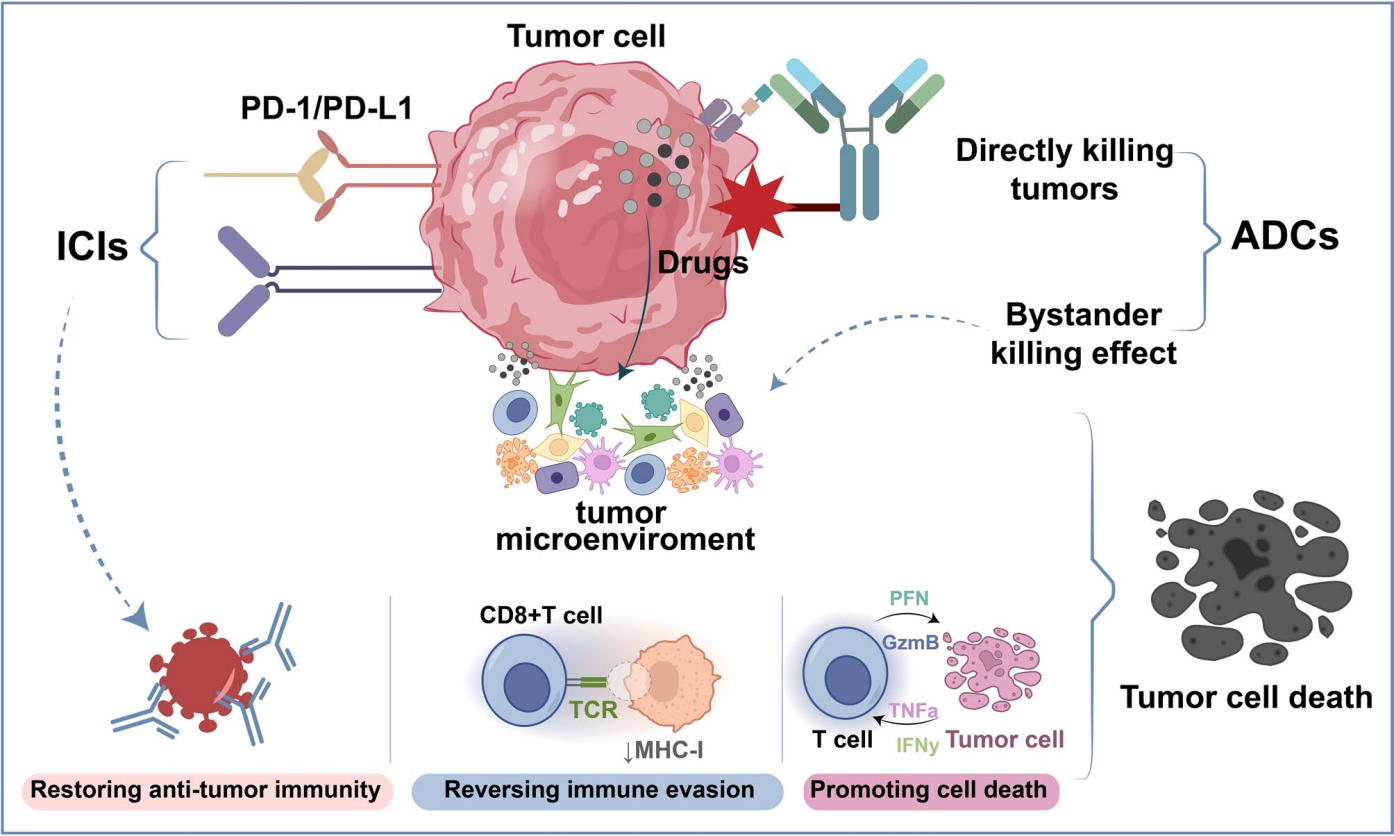
Combination of ICIs and ADCs for NSCLC Treatment. ICIs, including PD-1/PD-L1 and CTLA-4 blockers, potentiate innate antitumor immunity by disrupting immune evasion, restoring T-cell function, and inducing tumor cell death. ADCs eradicate tumors through dual mechanisms. First, antibody-mediated targeting of TSAs delivers cytotoxic payloads directly to malignant cells. Second, the bystander effect of ADCs extends cytotoxicity to adjacent tumor cells. These combined actions synergistically remodel the TME, achieving potent tumor eradication

ADCs are innovative therapeutic agents integrating three key components: specialized linkers, monoclonal antibodies targeting specific antigens and potent small-molecule cytotoxic drugs (Fig. 7) [167]. This design merges the precision of antibody-based tumor targeting with the potent cell-killing effects of conventional chemotherapy [168]. Upon intravenous administration, ADCs selectively bind to antigens on cancer cell surfaces. After antigen-mediated endocytosis, ADCs are internalized and transported to lysosomes. In lysosomes, ADCs undergo degradation to release the cytotoxic payload in their active form, which ultimately induces cancer cell death [169]. ADCs primarily exert antitumor effects through two mechanisms. The first involves internalizing the ADC-antigen complex into tumor cells via the endolysosomal pathway, which leads to the intracellular release of the cytotoxic payload and subsequent cell death [170]. Known as bystander killing, the second mechanism enables the diffusion of the released payload into neighboring cells, which induces apoptosis and enhances overall antitumor response. This dual-action approach strengthens the therapeutic efficacy of ADCs while minimizing off-target effects [171].

ADCs are combined with immunotherapy through diverse mechanisms, including Fc-mediated effector functions, the induction of immunogenic cell death (ICD) and direct DC activation and maturation. Firstly, the antibody component of ADCs delivers cytotoxic payloads and modulates innate immune responses through its Fc region. The Fc region interacts with Fcγ receptors (FcγRs) on immune cells like NK cells and macrophages [172]. This interaction mediates antibody-dependent cell-mediated cytotoxicity and phagocytosis (ADCC and ADCP). Immunoglobulin G1 (IgG1) is the most commonly used antibody subclass in ADCs by virtue of its long half-life and strong FcγR binding capacity [173]. However, Fc-mediated effector functions may produce side effects like thrombocytopenia caused by trastuzumab emtansine (T-DM1) binding to FcγRIIA on megakaryocytes. To optimize the immunomodulatory functions of ADCs, Fc regions can be engineered, for example, by introducing mutations to reduce FcγR binding or producing afucosylated IgGs to enhance ADCC, which thereby minimizes toxicity [174]. Secondly, ICD is a regulated cell death process triggered by ER stress and changes in cell surface composition. It is accompanied by the release of damage-associated molecular patterns (DAMPs), activating the immune system and establishing immunological memory [175, 176]. ICD can be induced by certain chemotherapeutic agents (e.g., anthracyclines and oxaliplatin) and cytotoxic payloads in ADCs (e.g., maytansine, pyrrolobenzodiazepine and microtubule inhibitors), which enhances anti-tumor immune responses. For instance, an ADC with an anthracycline derivative payload (T-PNU) substantially increased the expression of DAMPs in a breast cancer model and synergized with anti-PD-1 therapy to improve efficacy [177]. Additionally, ADCs like brentuximab vedotin and ladiratuzumab vedotin exhibit immunomodulatory effects induced by ICD, which further enhances the efficacy of ICIs [178, 179]. Thirdly, mature DCs are paramount in tumor immunity by the activation of anti-tumor T cell responses via the MHC class II complex [180]. Nevertheless, cancer cells generally evade immune surveillance through the inhibition of DC maturation or the induction of dysfunction. Microtubule-disrupting agents (e.g., maytansinoids and auristatins) can directly trigger the activation and maturation of DCs. It is a phenomenon particularly evident in immunocompetent animal models [181, 182]. For example, trastuzumab deruxtecan (T-DXd), a human epidermal growth factor receptor 2 (HER2)-targeted ADC carrying the topoisomerase I inhibitor DXd, significantly increased the number of DCs infiltrating tumors and markers of maturation and activation. Meanwhile, it enhanced the infiltration of CD8^+^ T cells and the expression of PD-L1 and MHC class I molecules on tumor cells [183].

Emerging evidence indicates that ADCs combined with immunotherapeutic agents exhibit enhanced sensitivity [184]. This synergy has spurred a growing interest in the integration of ADCs with immunotherapy in clinical settings. Preclinical research and early-phase clinical trials have demonstrated that such combinations can greatly amplify anti-tumor efficacy, which marks a promising direction in cancer treatment strategies. A phase Ib, multicenter trial evaluated whether the combination of T-DXd (DS-8201, a HER2-targeted ADC) and pembrolizumab in NSCLC patients was safe, tolerable and preliminarily effective [185]. The study was aimed at exploring the synergistic potential of these agents by leveraging the tumor-targeting and cytotoxic effects of T-DXd alongside the immune-activating properties of pembrolizumab. The combination was generally well-tolerated, with manageable adverse events such as fatigue, nausea and hematologic toxicities. Preliminary data on efficacy showed promising response rates in HER2-expressing NSCLC, including tumors with low HER2 levels. Biomarker analyses suggested relationships between HER2 expression, tumor mutation burden (TMB), immune infiltration and treatment responses, which provided insights for the selection of patients. These findings underline the potential of combining ADCs with ICIs to enhance therapeutic efficacy in advanced cancers and support further investigation in larger trials and the development of personalized treatment strategies.

**Fig. 8:**
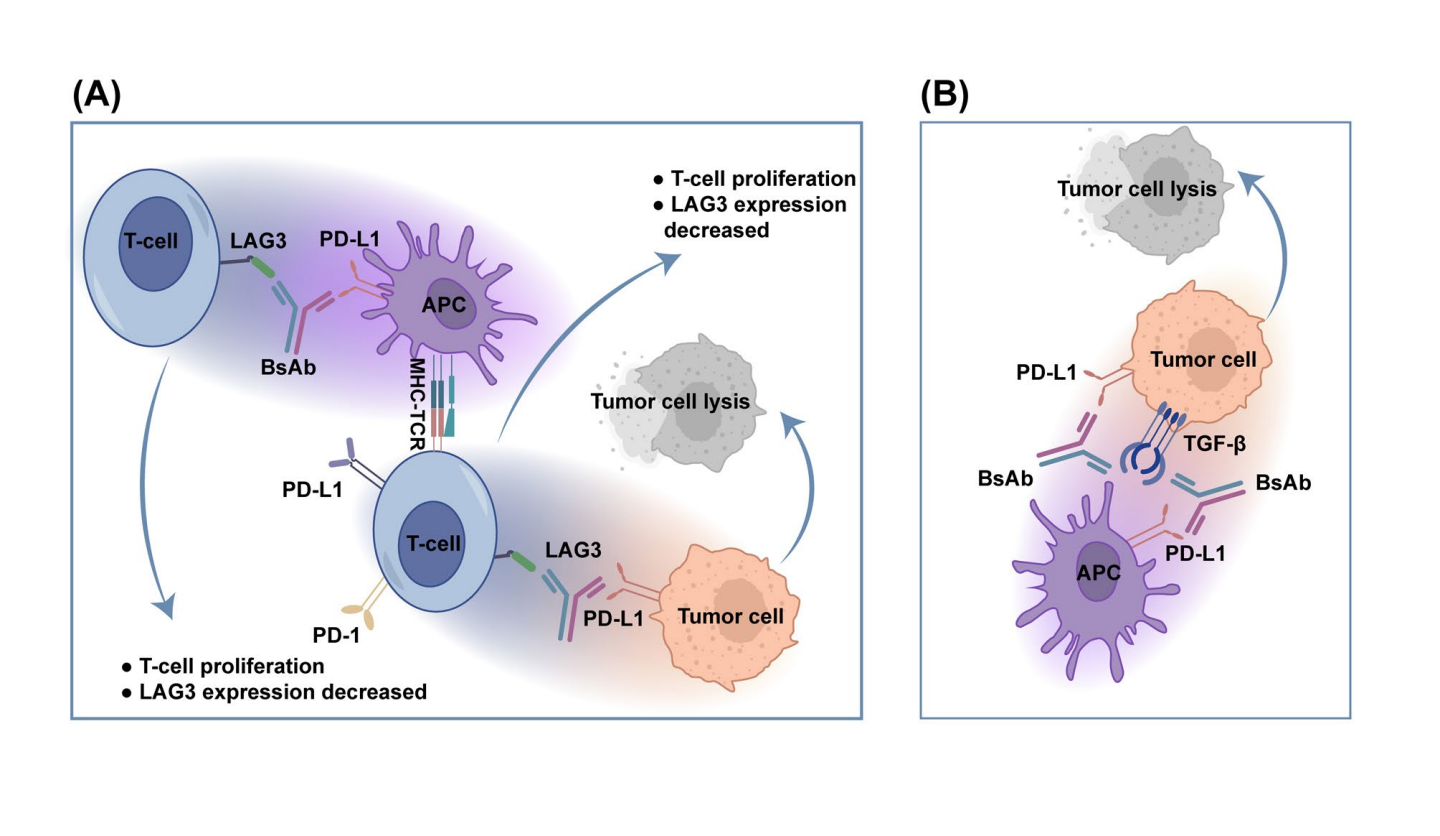
bsAbs in Combating Immunotherapy Resistance. (**A**) Dual ICP-blocking bsAbs co-engage LAG3 on T cells and PD-L1 on tumor cells/APCs, enhancing T cell activation. This triggers perforin/granzyme release, inducing tumor lysis and downregulating LAG-3 expression. (**B**) BsAb targets ICP concurrently with a molecule (e.g., TGF-β) involved in other signaling pathways

As antibody molecules constructed through genetic recombination, chemical conjugation or quadroma technology, bsAbs contain two distinct binding units, each of which is capable of recognizing specific epitopes independently (Fig. 8) [186, 187]. Up to now, seven bsAbs have received regulatory approval for hematologic malignancies, and four have been approved for specific solid tumors. This demonstrates the significant clinical efficacy of bsAbs [188]. Dual ICP-targeting bsAbs are designed to bind two distinct immune checkpoint receptors simultaneously. One arm targets an inhibitory receptor (e.g., PD-1, CTLA-4, LAG-3 and TIGIT) on T cells, while the other engages another receptor on T cells, tumor cells or APCs. This dual targeting leverages the co-expression of ICP, like CTLA-4 and PD-1, often on TILs, which is less common in peripheral T cells [189]. By preferentially targeting TILs through avidity-mediated selection, volrustomig (PD-1 + CTLA-4) [190], cadonilimab [191], and other bsAbs enhance the immunological synapse in the intercellular space. This potentially reduces on-target off-tumor toxicity and amplifies antitumor immune responses. A key advantage of bsAbs lies in their capability of mitigating adaptive resistance, which usually arises from the compensatory up-regulation of alternative ICP pathways after single-target blockade [192]. For instance, FS118 (F-star 118, LAG-3 + PD-L1) has been proven to reduce the expression of LAG-3 on T cells in preclinical models, whereas single or combined monoclonal antibodies aimed at LAG-3 or PD-L1 lead to increased LAG-3 expression. This suggests that bsAbs are more effective in disrupting inhibitory signals by facilitating the degradation and internalization of the two targets, which prevents compensatory up-regulation and alters T cell signaling dynamics. Beyond ICP blockade, BsAbs can also get involved in co-stimulatory receptor signaling via immunoglobulin B7-CD28 family (inducible co-stimulator (ICOS) or CD28) or tumor necrosis factor (TNF) superfamily members (CD27, CD40, tumor necrosis factor receptor superfamily, member 4 (OX40) and tumor necrosis factor receptor superfamily member 9 (4-1BB)). However, achieving a safe and effective balance in the stimulation of T cells remains a challenge, as excessive activation can provoke adverse effects [193]. Beyond that, bsAbs can redirect immune cells by targeting antigens involved in the progression, angiogenesis, metastasis and proliferation of tumors. For example, Ivonescimab (AK112, PD-1 + VEGF) combines vascular endothelial growth factor (VEGF) blockade in the TME with PD-1 immunomodulation, and demonstrates enhanced activity in NSCLC [194]. AK104 (PD-1/CTLA-4 bispecific antibody) in combination with anlotinib showed favorable antitumor activity and an acceptable safety profile in treatment-naïve patients with PD-L1 TPS ≥ 1% NSCLC [195]. Other investigational approaches encompass dual ICP blockade with molecules targeting immunosuppressive pathways, like TGF-β and PD-1/PD-L1, are being studied at present [196, 197]. As a whole, bsAbs offer a multifaceted approach to overcoming resistance to immunotherapy by enhancing precision, reducing toxicity and addressing compensatory resistance mechanisms. Future research may attach importance to optimizing these agents, exploring novel combinations and identifying biomarkers to guide their clinical applications, which ultimately advances the field of immuno-oncology.

## Predictive biomarkers and resistance monitoring in NSCLC immunotherapy

The accurate prediction of resistance to immunotherapy via biomarkers is pivotal for developing personalized treatment strategies, improving survival outcomes and minimizing potential risks. These biomarkers provide insights into the resistance mechanisms involving genetic, proteomic, epigenetic and metabolomic characteristics, and may reveals the complex interplay between the TME and immune system [198].

In the clinical management of NSCLC patients, PD-L1 and TMB are currently the two most extensively studied tissue-derived biomarkers. PD-L1 is the sole companion diagnostic biomarker widely applied in clinical decision-making. Guidelines from the National Comprehensive Cancer Network (NCCN) and the European Society for Medical Oncology (ESMO) recommend that PD-L1 be used as a primary determinant for immunotherapy selection in metastatic NSCLC: patients with high PD-L1 expression (TPS ≥ 50%) are eligible for anti-PD-1/PD-L1 monotherapy, while those with intermediate expression (TPS 1–49%) are advised to receive combination chemo-immunotherapy [199]. Nevertheless, PD-L1 remains to be controversial as a predictive biomarker. A systematic review of 45 immunotherapy trials approved by the FDA found that PD-L1 had clear predictive value in only about 29% of the studies, while no correlation was detected in 53% of the studies [200]. This mirrors the limitation in predictive accuracy because of the dynamic expression, spatial heterogeneity and methodological variability across PD-L1 detection platforms. As another predictive biomarker, higher TMB correlates with elevated neoantigen load, thereby boosting tumor immunogenicity [17, 201]. However, TMB also has its limitations as a standalone biomarker: high TMB does not necessarily translate into therapeutic benefits, nor does low TMB exclude potential responders. Furthermore, TMB testing lacks standardization. Inconsistencies arise from discrepancies in panel coverage, mutation-counting criteria, and sample quality across sequencing platforms [202]. The FDA-recommended threshold of ≥ 10 mut/Mb has also been questioned in several studies [198].

With the emergence of liquid biopsies in diagnostics, circulating biomarkers have shown increasing promise in immunotherapy for NSCLC. Circulating tumor DNA (ctDNA) is a most extensively studied biomarker for liquid biopsies [203]. It reflects genomic mutations, clonal evolution and tumor burden. In addition, ctDNA can be sensitively quantified using technologies like next-generation sequencing (NGS) and droplet digital polymerase chain reaction (ddPCR) [204]. Multiple studies have shown that significant post-treatment declines in ctDNA levels are closely associated with improved ORR, PFS and OS in NSCLC patients receiving ICIs [205]. Conversely, persistently high or insufficiently reduced ctDNA levels typically indicate poor immunotherapy responses and may signal disease progression or early relapse. For instance, a meta-analysis of over 1,000 NSCLC patients revealed no significant association between pre-treatment ctDNA and immune responses. However, ctDNA decline after treatment was strongly correlated with improved PFS and OS [206]. Moreover, ctDNA clearance has been proposed as an early indicator of treatment responses. It is closely linked to pathological complete response (pCR) in neoadjuvant ICI settings [207].

Beyond ctDNA, tumor-derived exosomal PD-L1 (exoPD-L1) has emerged as a hot liquid biopsy biomarker [198]. As critical intercellular mediators, exosomes may carry functional molecules like PD-L1 protein and microRNAs that suppress T cell activation and promote immune evasion. Studies have suggested that NSCLC patients with higher baseline exoPD-L1 levels are inclined to have poorer responses to immunotherapy, along with shorter PFS and OS [208]. However, the conflicting results across studies probably stem from differences in sample processing, detection platforms and the varied cellular origins of exoPD-L1 [209]. The lack of standardization in the detection of exosomes and limited biological validation remain major barriers to its clinical translation.

Peripheral immune components, notably MDSCs and circulating T cells, critically influence ICI response and resistance. In NSCLC, clinical outcomes correlate with peripheral CD8⁺ T-cell phenotypes, including activation status, exhaustion markers, clonality, etc [210]. High-frequency effector or memory-like circulating CD8⁺ T cells correlate with durable responses, whereas the expansion of exhausted or Treg populations may indicate emerging resistance [211]. Moreover, MDSCs in peripheral blood may suppress T cell responses by depleting essential metabolites (e.g., arginine), producing ROS or secreting IL-10, TGF-β and other immunosuppressive cytokines [212]. Elevated baseline levels of circulating MDSCs have been linked to poor response and shorter PFS following ICI therapy. Therefore, profiling peripheral immune signatures offers a low invasive strategy for forecasting immunotherapy resistance. It may guide the development of combinatorial approaches to overcome systemic immune suppression.

Meanwhile, increasing circulating immune parameters are being integrated into predictive models for immunotherapy. Studies have shown that the immune activation status of the host is reflected by the absolute numbers of peripheral CD8⁺ and CD4⁺ T lymphocytes, changes in Treg proportions, and the diversity and clonal expansion of the TCR repertoire [203]. The diversity of TCRs is considered to mark the ability of the immune system to recognize tumor neoantigens. Broad TCR repertoires before treatment, together with increased diversity during therapy, are usually associated with better ICI responses and long-term survival [213]. Another commonly used indicator is the neutrophil-to-lymphocyte ratio (NLR). As an indirect marker of systemic inflammation, NLR has been confirmed by multiple studies to have a connection with immunotherapy prognosis. As a general rule, higher NLR values suggest a state of chronic inflammation and immunosuppression, which reflects an unfavorable TME. Therefore, they are closely linked to unfavorable PFS and OS [198]. However, no consensus has been reached on the optimal cutoff value for NLR, which limits its widespread clinical application.

Beyond circulating biomarkers, gene expression features at the transcriptomic level have also turned into important tools for predicting immunotherapy responses. For the purpose of comprehensively characterizing the composition, activation status and pathway activity of various cell types within the TME, researchers have developed numerous immune-related gene signatures on the basis of targeted or global RNA-seq data [214]. Often enriched for gene modules related to T cell activation, cytotoxicity, IFN signaling and DDR, these signatures have been found in several studies to predict immunotherapy benefits and survival outcomes in NSCLC patients. Conversely, tumors from ICI non-responders typically exhibit activated stromal remodeling, immunosuppression, and angiogenesis pathways, defining a ‘cold’/immune-excluded TME molecular phenotype. For instance, transcriptomic signatures that are enriched in genes related to cytolytic activity, T cell activation, IFN signaling and DDR can predict the efficacy and survival outcomes of immunotherapy [214, 215]. However, the transcriptomes of ICI non-responders exhibit up-regulated genes associated with stromal remodeling, immunosuppression and angiogenesis [216]. In the past few years, spatial transcriptomics has rapidly emerged as a key complement to bulk RNA-seq, enabling transcriptome mapping at single-cell or regional resolution while preserving tissue architecture [217, 218]. A recent retrospective study of NSCLC tumors and adjacent tissues with spatial transcriptomics revealed robust B-cell activation and B-cell-mediated immune pathways in ICI responders, supporting the important regulatory role of B cells in ICI responsiveness [217].

By and large, biomarkers hold transformative potential for deciphering immunotherapy resistance mechanisms and enabling personalized efficacy prediction, with clinical applications in early decision making, treatment monitoring, and stratified therapy. Despite promise, key biomarkers, including ctDNA, exoPD-L1, and immune cell parameters, remain exploratory due to unstandardized detection platforms/criteria and analytical validation gaps. Future efforts must prioritize large-scale prospective validation of the sensitivity/specificity and clinical utility of respective biomarkers while driving standardization and commercialization of detection technologies to realize precision immuno-oncology.

## Challenges and future perspectives

The clinical success of ICIs in NSCLC has redefined treatment paradigms, yet primary and acquired resistance remain persistent challenges. Resistance arises from both tumor-intrinsic factors (e.g., neoantigen loss, genetic mutations, epigenetic plasticity) and tumor-extrinsic components within the TME, such as suppressive cytokines, Tregs, MDSCs, TANs and TAMs. These mechanisms collectively disrupt immune surveillance and enable immune escape. Recent findings suggest that resistance extends beyond the TME. Peripheral immune exhaustion, dysfunctional myeloid trafficking, systemic inflammation, and microbiota-derived metabolites all modulate ICIs efficacy, underscoring the insufficiency of a TME-centric view. Interpatient heterogeneity in HLA genotype, baseline immune tone, and microbial ecology further complicates response prediction and highlights the absence of universal biomarkers.

Overcoming these challenges demands systems-level strategies with spatiotemporal immune profiling. Single-cell RNA-seq, spatial transcriptomics, and multi-omics empower high-resolution mapping of immune dynamics and resistance evolution. These tools can identify predictive and dynamic biomarkers for real-time monitoring and precision interventions.

Future NSCLC regimens must evolve beyond conventional ICIs monotherapy. Rational combinations, incorporating chemotherapy, RT, HDAC/BET inhibitors, ADCs, bsAbs, and small-molecule immunomodulators, should leverage dynamic immune contexture and resistance mechanisms. Adjunct strategies like microbiome modulation, metabolic reprogramming, and mRNA vaccines warrant clinical evaluation. Ultimately, advanced NSCLC immunotherapy requires the integration of precision medicine, systems immunology, and adaptive trial design. By co-targeting local/systemic resistance axes, durable responses in non-responders may be achievable. Multidimensional biomarker-driven approaches are essential to overcome resistance and achieve long-lasting benefit of precision immuno-oncology.

## Data Availability

No datasets were generated or analysed during the current study.

## Abbreviations

ACT: Adoptive Cellular Therapy
ADCs: Antibody-Drug Conjugates
APCs: Antigen Presenting Cells
BETis: Bromodomain and Extra-Terminal Motif Inhibitors
bsAbs: Bispecific Antibodies
CAR-T: Chimeric Antigen Receptor T Cell
cGAS-STING: Cyclic GMP-AMP Synthase-Stimulator of Interferon Genes
CTLA-4: Cytotoxic T-Lymphocyte-Associated Protein 4
DCs: Dendritic Cells
EGFR: Epidermal Growth Factor Receptor
FDA: Food and Drug Administration
FcγR: Fc Gamma Receptor
HDACis: Histone Deacetylase Inhibitors
HLA: Human Leukocyte Antigen
ICD: Immunogenic Cell Death
ICIs: Immune Checkpoint Inhibitors
ICOS: Inducible Co-stimulator
IDO1: Indoleamine 2,3-Dioxygenase 1
ICP: Immune Checkpoint
IFN-γ: Interferon Gamma
IgG1: Immunoglobulin G1
ILs: Interleukins
LAG-3: Lymphocyte Activation Gene 3
MDSCs: Myeloid-Derived Suppressor Cells
MHC: Major Histocompatibility Complex
NSCLC: Non-Small Cell Lung Cancer
PD-1: Programmed Cell Death Protein 1
PD-L1: Programmed Death-Ligand 1
PFS: Progression Free Survival
PRRs: Pattern Recognition Receptors
ROS: Reactive Oxygen Species
SHIP1: SH2-containing Inositol-5’-Phosphatase 1
TANs: Tumor-Associated Neutrophils
TDO: Tryptophan 2,3-Dioxygenase
TIGIT: T-cell Immunoreceptor with Ig and ITIM domains
TILs: Tumor-Infiltrating Lymphocytes
TIM-3: T-cell Immunoglobulin and Mucin-domain containing-3
TLR3: Toll-Like Receptor 3
TMB: Tumor Mutational Burden
TME: Tumor Microenvironment
TNF-α: Tumor Necrosis Factor -alpha
Tregs: Regulatory T Cells
VEGF: Vascular Endothelial Growth Factor
